# Determinants of high residual post-PCV13 pneumococcal vaccine-type carriage in Blantyre, Malawi: a modelling study

**DOI:** 10.1186/s12916-019-1450-2

**Published:** 2019-12-05

**Authors:** J. Lourenço, U. Obolski, T. D. Swarthout, A. Gori, N. Bar-Zeev, D. Everett, A. W. Kamng’ona, T. S. Mwalukomo, A. A. Mataya, C. Mwansambo, M. Banda, S. Gupta, N. French, R. S. Heyderman

**Affiliations:** 1 Department of Zoology, University of Oxford, Oxford, UK; 2 School of Public Health, Tel Aviv University, Tel Aviv, Israel; 3 Porter School of the Environment and Earth Sciences, Tel Aviv University, Tel Aviv, Israel; 4 Malawi-Liverpool-Wellcome Trust Clinical Research Programme, Blantyre, Malawi; 5 Clinical Sciences Department, Liverpool School of Tropical Medicine, Liverpool, UK; 6 NIHR Mucosal Pathogens Research Unit, Division of Infection & Immunity, University College London, London, UK; 7 Department of International Health, Johns Hopkins Bloomberg School of Public Health, Baltimore, USA; 8 The Queens Medical Research Institute, University of Edinburgh, Edinburgh, UK; 9 Department of Biomedical Sciences, College of Medicine, University of Malawi, Blantyre, Malawi; 10 Department of Medicine, College of Medicine, University of Malawi, Blantyre, Malawi; 11 Ministry of Health, Lilongwe, Malawi; 12 Ministry of Education, Blantyre, Malawi; 13 Centre for Global Vaccine Research, Institute of Infection and Global Health, University of Liverpool, Liverpool, UK

**Keywords:** Pneumococcus, pcv13, Modelling, Malawi, Intervention

## Abstract

**Background:**

In November 2011, Malawi introduced the 13-valent pneumococcal conjugate vaccine (PCV13) into the routine infant schedule. Four to 7 years after introduction (2015–2018), rolling prospective nasopharyngeal carriage surveys were performed in the city of Blantyre. Carriage of *Streptococcus pneumoniae* vaccine serotypes (VT) remained higher than reported in high-income countries, and impact was asymmetric across age groups.

**Methods:**

A dynamic transmission model was fit to survey data using a Bayesian Markov-chain Monte Carlo approach, to obtain insights into the determinants of post-PCV13 age-specific VT carriage.

**Results:**

Accumulation of naturally acquired immunity with age and age-specific transmission potential were both key to reproducing the observed data. VT carriage reduction peaked sequentially over time, earlier in younger and later in older age groups. Estimated vaccine efficacy (protection against carriage) was 66.87% (95% CI 50.49–82.26%), similar to previous estimates. Ten-year projected vaccine impact (VT carriage reduction) among 0–9 years old was lower than observed in other settings, at 76.23% (CI 95% 68.02–81.96%), with sensitivity analyses demonstrating this to be mainly driven by a high local force of infection.

**Conclusions:**

There are both vaccine-related and host-related determinants of post-PCV13 pneumococcal VT transmission in Blantyre with vaccine impact determined by an age-specific, local force of infection. These findings are likely to be generalisable to other Sub-Saharan African countries in which PCV impact on carriage (and therefore herd protection) has been lower than desired, and have implications for the interpretation of post-PCV carriage studies and future vaccination programs.

## Background

*Streptococcus pneumoniae* (pneumococcus) is a bacterial human pathogen commonly carried asymptomatically in the nasopharynx, which in a minority of carriers can cause severe disease such as pneumonia, meningitis or bacteremia [1], posing a serious mortality risk, especially for young children (< 5 years of age), the elderly (> 65 years of age) and the immunocompromised [2]. Pneumococcal carriage is a necessary precursor of severe disease [3] and transmission, such that reduction of carriage through active control is an important, universal public health goal.

Currently, pneumococcal conjugate vaccines (PCV) are the best available tool to reduce carriage and disease both within risk groups and the general population. These vaccines have consisted of either 7, 10 or 13 polysaccharides conjugated to a carrier protein (PCV7, PCV10, PCV13, respectively). All have been demonstrated to be highly protective against 7, 10 or 13 common pneumococcal serotypes associated with carriage and disease (also termed vaccine serotypes, VT). A frequently observed consequence of PCV introduction is the increase in both carriage and disease of non-VT pneumococci (NVT), likely due to increased niche availability and reduction of competition between VT and NVT [4–9].

PCV routine vaccination has been a common control strategy for over a decade in developed countries, with past experience showing that both pre- and post-PCV pneumococcal carriage can be highly variable within and between countries [10–16]. PCV vaccines have only recently been introduced in sub-Saharan African countries, such as Kenya [17, 18], Malawi [19], The Gambia [20] and South Africa [21]. In November 2011, Malawi introduced the 13-valent pneumococcal conjugate vaccine (PCV13) as part of the national extended program of immunisation with a 3 + 0 schedule (at 6, 10 and 14 weeks of age). With high routine coverage (~ 90%) and a small catch-up campaign of young children, PCV13 was expected to quickly reduce carriage as previously reported in developed countries. However, recently published data on nasopharyngeal carriage as measured in a cross-sectional observational study in Blantyre (Southern Malawi), 4 to 7 years after PCV13 introduction (2015–2018), has shown that vaccine impact (VT carriage reduction) has been slower than expected and heterogeneous across age groups [22]. Epidemiological mathematical models have previously been employed successfully to improve our understanding of pneumococcal dynamics [5, 9, 23–27], as well as having contributed to explain, estimate and project PCV impact [8, 11, 28]. The main advantage of models is their cost-free potential to test hypotheses and gain a mechanistic, ecological and immunological understanding of carriage and disease dynamics, estimating epidemiological parameters which are difficult to otherwise quantify from raw epidemiological data. For example, models have successfully yielded estimates of VT and non-VT pneumococci transmission potentials [26, 29–31], pneumococcal competition factors [8, 9, 23, 28, 32, 33] and measures of vaccine-induced protection from carriage at the individual level [11, 17, 28, 34, 35], none of which are readily observed or quantified in cross-sectional observational studies.

In this study, we use a Bayesian Markov chain Monte Carlo fitting approach and a dynamic model to investigate the post-PCV13 pneumococcal VT carriage dynamics in Blantyre, Malawi. We find that natural immunity and age-specific transmission potentials are necessary to reproduce observed VT carriage. When compared to numerous reports in the literature from other regions, our estimated vaccine efficacy (individual-level protection from carriage) was close to expected values, but impact (population-level reduction of VT carriage) was lower both in the short and long term. We show that vaccine impact was likely being offset by a high local force of infection compared to other regions of the world. Our study offers new insights into the lower than expected PCV13 impact in Malawi and more generally on the heterogeneous nature of pre- and post-vaccination pneumococcal VT carriage across age groups and regions. These results can be translated to other sub-Saharan African countries in which PCV impact and herd protection has been lower than desired.

## Methods

### Prospective cross-sectional observational study

**Fig. 1:**
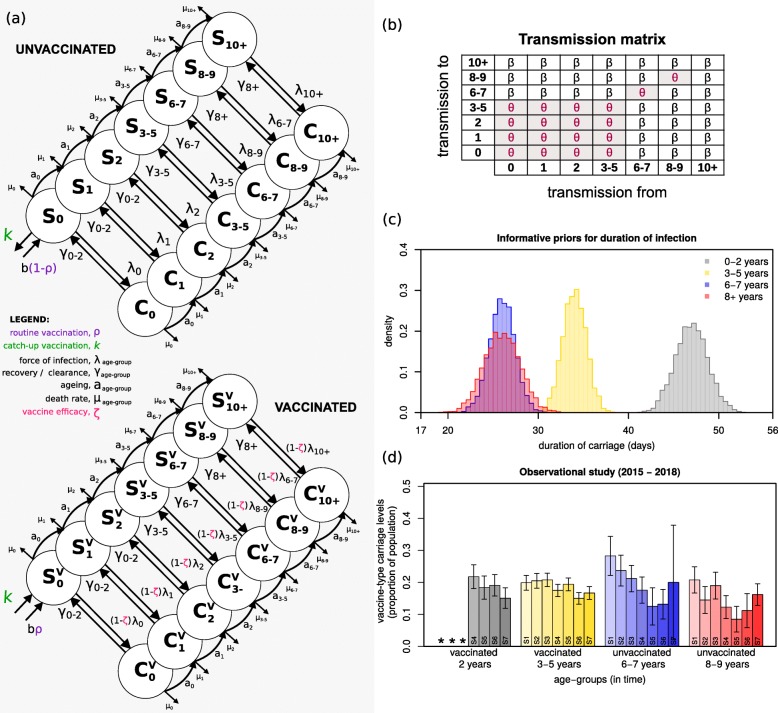
Survey data and model framework, priors and transmission matrix. **a** Seven age groups were modelled: 0, 1, 2, 3–5, 6–7, 8–9, 10+ years of age (circles), each divided into unvaccinated (top) and vaccinated (bottom). Labels *a*_age group_ mark ageing rates per age class; *μ*_age group_ mark age-specific death rates; b marks births, at which point a proportion (*ρ*) are vaccinated (purple); *ζ* marks vaccine-induced protection, expressed as reduction in susceptibility to infection of vaccinated individuals (magenta); *λ*_age group_ mark age-specific forces of infection; *γ*_age group_ mark age-specific rates of clearance from infection; k marks catch-up vaccination (green). **b** The transmission matrix used, with coefficients *β* and *θ*, where *θ* is the specific coefficient for transmission within and between particular age groups. *β* and *θ* are estimated when fitting the survey data. **c** The informative priors used in the fitting exercise for mean (standard deviation) infectious periods (days) of 47 (1.8) for 0–2 years old; 34 (1.3) for 3–5 years old; 26 (1.4) for 6–8 years old; 26 (2.0) for 8+ years old. The posterior values of these periods (1/*γ*_0–2_, 1/*γ*_3–5_, 1/*γ*_6–8_, 1/*γ*_8+_) are estimated when fitting the survey data. **d** Mean and standard error for carriage as reported in the observational study data (surveys) per age group (Additional file 1: Table S7). S1 to S7 highlight the surveys 1 to 7. The * mark data that was not collected

An observational study using stratified random sampling was conducted to measure pneumococcal nasopharyngeal carriage in Blantyre, Malawi [22]. Sampling was performed twice a year, between June and August 2015 (survey 1), October 2015 and April 2016 (survey 2), May and October 2016 (survey 3), November 2016 and April 2017 (survey 4), May and October 2017 (survey 5), November 2017 and June 2018 (survey 6) and June and December 2018 (survey 7). In this study, we use the mid-point dates of the surveys for model fitting and presentation of results. A total of 7148 individuals were screened with nasopharyngeal swabs processed following WHO recommendations [36]. Isolates were serotyped by latex agglutination (ImmuLex™ 7-10-13-valent Pneumotest; Statens Serum Institute, Denmark). In this study, we use all the data from three age groups: 499 vaccinated children 2 years old, 2565 vaccinated children 3–7 years old and 1402 unvaccinated children 3–10 years old. For the first three surveys, data on vaccinated 2 years old individuals was not collected. Observed VT carriage levels are presented in Fig. 1d and Additional file 1: Table S7. Further details on collection, processing and observations, as well as the dynamics of non-VT have been previously described in detail [22].

### Vaccine type transmission model

A deterministic, ordinary-differential equations (ODE) model (Fig. 1a) was developed to fit VT carriage levels as reported in the cross-sectional observational study in Blantyre (Fig. 1d) [22]. Fitting was implemented using a Bayesian Markov chain Monte Carlo (bMCMC) approach developed and used by us in other modelling studies [37–39], including informative priors for duration of carriage (Fig. 1c) and uninformative uniform priors for vaccine efficacy (individual-level protection against carriage) and transmission potential. The bMCMC searches the parameter space for combinations that result in pre-vaccination VT carriage levels which, when affected by the introduction of the vaccine, result in dynamics similar to those observed in the post-vaccination era. Thus, although the approach does not use pre-vaccination VT carriage data, it can still estimate the most likely combination of pre-vaccination carriage and vaccine effects that leads to observed, post-vaccination dynamics. The methodology is summarised in this section and further details can be found in Additional file 1, such as equations, literature review on priors and expected parameter values and complementary results.

### Pneumococcal infection dynamics and human demographics

As depicted in Fig. 1a, the population was divided into seven non-overlapping age groups: 0 (< 1), 1, 2, 3–5, 6–7, 8–9, 10+ years old. Ageing was approximated by moving individuals along age groups with a rate (*a*_age group_) equal to the inverse of the time spent at each age class. The seven age groups were further divided into vaccinated (*S*^*v*^_age group_, *C*^*v*^_age group_) and unvaccinated (*S*_age group_, *C*_age group_) susceptibles (*S*) and carriers (*C*). The population size was assumed to be constant, with total deaths equal to births (details in Additional file 1). Death rates were age-specific (*μ*_age group_) and relative to a generalised total lifespan of 70 years.

### Natural immunity

Pneumococcal colonisation increases both humoral (anti-capsular serotype-specific and anti-protein non-serotype-specific) and T cell (anti-protein) immunity [40]. Acquisition of this immunity correlates with colonisation in children and increases with age as colonisation decreases. In our model (Fig. 1a), all individuals were assumed to be born susceptible but can acquire infection (colonisation) at any age with a particular force of infection *λ*_age group_, becoming carriers (*C*_age group_) for an age-specific period (1/*γ*_age group_), and returning to the susceptible state (*S*_age group_) after clearance. Hence, the development of complete (sterile) immunity to the pneumococcus was not considered. We nonetheless allowed for decreasing duration of carriage with age (1/*γ*_age group_) as a proxy for the development of pneumococcal immunity with age. To quantify differences in age, we used carriage duration data as reported by Hogberg and colleagues [41] to define informative priors related to the aggregated age groups: 0–2 years (1/*γ*_0–2_), 3–5 years (1/*γ*_3–5_), 6–8 years (1/*γ*_6–8_) and 8+ years (1/*γ*_8+_) as represented in Fig. 1c (Additional file 1: Table S1 for literature review).

### Vaccination, efficacy and impact

For simplicity, routine vaccination was implemented at birth with coverage (*ρ*) at 92.5% [22], and catch-up implemented as a one-off transfer of a proportion of individuals from the unvaccinated susceptibles with 0 (< 1) years of age (*S*_0_) to the vaccinated susceptible class with the same age (*S*^*v*^_0_) with coverage of 60% (at time of vaccine introduction) [22]. We assumed the vaccine to reduce the risk of infection (colonisation) of vaccinated individuals by a proportion *ζ* (between 0 and 1, with *ζ* = 1 equating to no risk). This reduction in risk was herein defined and interpreted as the individual-level vaccine efficacy against carriage (VE = 100 × *ζ*) and was modelled directly on the force of infection (*λ*) (Fig. 1a and Additional file 1: Table S2 for literature review). We measured vaccine impact across age groups as the post-PCV13 percent reduction in population-level VT carriage compared to pre-vaccination levels.

### Force of infection

We considered several transmission matrices (Additional file 1) and compared the resulting model fits using leave-one-out cross-validation (LOO) and the widely applicable information criterion (WAIC) measures [42–44]. The inhomogeneous transmission matrix presented in Fig. 1b over-performed the others and was used for the results presented in the main text. Its structure is based on epidemiological studies conducted in American, European and African populations reporting typical, strong, intrinsic variation in frequency, efficiency and environmental risk of transmission between age groups [10, 31, 45–50]. In summary, the transmission matrix is generally populated with a baseline coefficient *β*, and a different coefficient *θ* assigned to transmission occurring within and between ages 0–5 years, and within 6–7 and 8–9 years of age independently. Further literature support and results from the second best-performing transmission matrix can be found in Additional file 1.

### Fitting to survey data

The model’s carriage outputs for vaccinated 2, vaccinated 3–5, unvaccinated 6–7 and unvaccinated 8–9 years of age were fitted to observed levels in Blantyre’s 1–7 surveys (Fig. 1d, values in Additional file 1: Table S7), approximately 4 to 7 years post PCV13 introduction (2015–2018). A total of seven parameters were fitted: vaccine efficacy against carriage (*ζ*, uninformative prior), coefficients of transmission (*β*, *θ*, uninformative priors) and durations of carriage in ages 0–2, 3–5, 6–7, 8+ years (1/*γ*_0–2_, 1/*γ*_3–5_, 1/*γ*_6–8_, 1/*γ*_8+_, informative priors). The transmission model was initialized at time *t* = 0 with a proportion of 0.99 susceptibles and 0.01 infected, with numerical simulations run until an equilibrium was reached. At equilibrium, vaccination was introduced and the first post-vaccine 15 years recorded. Levels of carriage in the model were calculated as the proportion of individuals within an age group that are carriers (i.e. *C/(S + C)*, expressions in Additional file 1). The model was run with parameters scaled per year. bMCMC chains were run for 5 million steps, with burn-in of 20% (bMCMC details in see Additional file 1).

## Results

We used our deterministic transmission model and bMCMC approach to fit the observed post-vaccination VT carriage data from Blantyre, Malawi (2015–2018). Based on this fit, we could reconstruct age-specific carriage dynamics for the unobserved first 4 years (2011–2015), and project VT carriage reduction into the future, to identify the mechanistic nature of the slow PCV13 impact on the vaccinated age groups and strong herd-effects in the older unvaccinated age groups.

### Model fit and posteriors

**Fig. 2:**
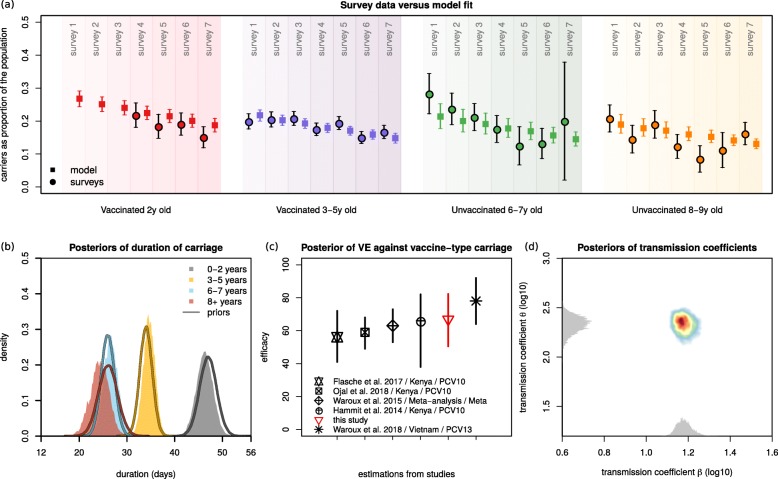
Model fit and estimated posteriors. **a** Model fit to carriage data from the observational study for different age groups: vaccinated 2 years old (red), vaccinated 3–5 years old (purple), unvaccinated 6–7 years old (green) and unvaccinated 8–9 years old (orange). The survey data is represented by full circles, the model output by full squares (data in Fig. 1d, Additional file 1: Table S7). **b** Priors (lines) and estimated posterior distributions (shaded) of duration of carriage per age group. **c** Visual comparison of the estimated mean and 95% CI of posterior of vaccine efficacy against vaccine-type carriage (red) in the context of estimates from other studies (in legend, Additional file 1: Table S2). **d** The estimated posterior distributions of the transmission coefficients *β* and *θ* are shown in two dimensions (coloured area). The estimated actual distribution for *β* is in the *x*-axis and *θ* in the *y*-axis (visualised in grey). Note that, for visualisation purposes, the axes are log_10_-transformed and the grey distributions’ height has no scale (height is not quantified). **a**–**d** Solutions presented are obtained from sampling 100,000 parameter values from posteriors and simulating the dynamic model

VT carriage levels across age groups reported from the surveys were closely reproduced by the mean and 95% CI of the model using the bMCMC approach (Fig. 2a). Our initial assumption of natural immunity accumulating with age was generally respected in the bMCMC solution (Fig. 2b); i.e. the estimated posterior distributions of the durations of carriage (1/*γ*_age group_) were adjusted by the bMCMC by approximately − 0.7, + 0.64, + 0.58 and − 1.73 days for the age groups 0–2, 3–5, 6–7 and 8+ years of age, respectively. The posterior distribution of vaccine efficacy (individual-level protection against carriage) across ages was estimated to be 66.87% (95% CI 50.49–82.26). While we used an uninformative prior (uniform, 0 to 1) in the bMCMC, this efficacy posterior was similar to others recently estimated with different models and in multiple epidemiological settings (Fig. 2c). We therefore argue that it serves as partial validation for our modelling framework. Finally, the solutions for the transmission coefficients *β* and *θ* suggested that in order to reproduce the Blantyre survey data, the risk of infection associated with contacts within and between younger age groups (0–5 years old) would have to be higher than that of the general population (i.e. *θ*>>*β*).

### Vaccine impact across age groups

**Fig. 3:**
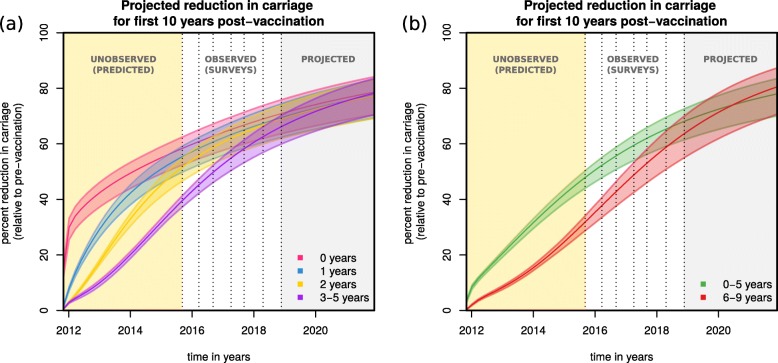
Projections of post-vaccination vaccine-type carriage reduction. **a** Projected reduction in carriage relative to the pre-vaccination era for age groups 0 years (magenta), 1 year (blue), 2 years (yellow) and 3–5 years (purple) old. **b** Projected reduction in carriage relative to the pre-vaccination era for aggregated age groups 0–5 years (green) and 6–9 years (red) old (with corresponding 95% CIs). **a**, **b** Solutions presented are obtained from sampling 100,000 parameter values from posteriors and simulating the dynamic model. The shaded areas are yellow for the post-vaccination period with no carriage data, white for the post-vaccination period with data, and grey for the post-vaccination projected period up to 10 years. Dotted vertical lines mark survey dates. The *x*-axis origin marks PCV13 introduction

Using parameter samples from the bMCMC estimated posteriors, we simulated vaccine impact in terms of VT carriage reduction across age groups in the first 10 years post-vaccination (Fig. 3).

After the first year, VT carriage reduction was estimated to be 42.38% (95% CI 37.23–46.01%) for the 0 (< 1) years old, followed by 29.25% (95% CI 26.4–31.4%) for the 1 year old, 17.45% (95% CI 16.47–18.36%) for the 2 years old and 4.95% (95% CI 8.78–10.89%) for 3–5 years old (Fig. 3a). With time, as carriage generally dropped and vaccinated individuals aged, the older groups were estimated to benefit from increasingly similar reductions in carriage compared to the initially vaccinated group. Since during the first year only the 0 (< 1) years of age were vaccinated, the short-term reductions in carriage of the other groups were due to indirect herd effects alone.

At the target point of 10 years into the post-vaccination era, impact was estimated to be similar across all age groups, with VT carriage reduced by 76.9% (CI 95% 68.93–82.32%) for the 0 (< 1) years old, 75.72% (CI 95% 67.78–81.24%) for the 1 year old, 75.51% (CI 95% 67.55–81.05%) for the 2 years old and 75.86% (CI 95% 68.29–80.97%) for 3–5 years old. We further projected vaccine impact on aggregated age groups 0–5 and 6–9 years of age, which showed equivalent reductions in VT carriage (Fig. 3b), with the larger aggregated age group 0–9 years old having a total reduction of 76.23% (CI 95% 68.02–81.96%) after 10 years.

We performed a literature review on observed reduction of VT carriage in time after the introduction of PCV vaccines (Additional file 1: Table S5) in numerous countries and concluded that both the observed carriage levels during the surveys and during the model’s projection for the first 10 years were high when compared to other countries. For instance, residual carriage of PCV13 types was 0.4% after 4 years of vaccination in England [51], 9.1% after 2 years of vaccination in Italy [52] and 7% after 3 years of vaccination in Alaska, USA [16]. Similarly, for 0–5-year-old individuals, PCV10 in Kenya [18] has reduced VT carriage by 73.92% in the first 5 years, while in Portugal [53], PCV7 has reduced VT carriage by 78.91% in the same age group and amount of time (more examples can be found on Additional file 1: Table S5).

### Post-vaccination changes in force of infection

To try to understand responses to vaccination across age groups, we further explored the post-PCV13 force of infection (FOI) dynamics. The FOI is the overall rate by which a certain age group of susceptible individuals is infected, comprising the transmission rate (*β* or *θ*) weighted by the number of infectious individuals within the same and other age groups. Although we modelled six independent age groups under 10 years of age, only three unique FOIs are defined in the transmission matrix for individuals under 9 years of age (0–5, 6–7 and 8–9 years of age, Fig. 1b).

**Fig. 4:**
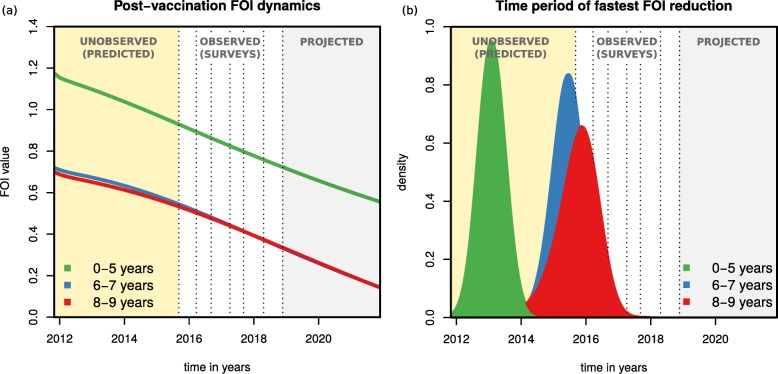
Projections of post-vaccination changes in the force of infection. **a** The post-vaccination force of infection (FOI) of different age groups (0–5 years in green, 6–7 in blue and 8–9 in red) as calculated for each of 100,000 simulations using parameter samples from posteriors. **b** For each FOI of each age group and each 100,000 simulations using parameter samples from posteriors, the time point of minimum derivative was calculated, resulting in one distribution per age group (coloured curves, 0–5 years in green, 6–7 in blue, 8–9 in red). This time point is as a proxy for the period of fastest FOI reduction. The shaded areas are yellow for the post-vaccination period with no carriage data, white for the post-vaccination period with data, and grey for the post-vaccination projected period up to 10 years. Dotted vertical lines mark survey dates. The *x*-axis origin marks PCV13 introduction

As determined by the posteriors of *β* and *θ* (Fig. 2d), the pre-vaccination absolute FOI of the 0–5, 6–7 and 8–9 age groups was different at PCV13 introduction, and with vaccine roll out the FOI of each age group decreased in time (Fig. 4a). We also examined the FOI derivative with respect to time as a measure of speed of FOI reduction (Fig. 4b) and found that the time period of fastest FOI reduction for the 0–5 years old was between vaccine introduction and 2015 (when no carriage data was collected). This contrasted with the older age groups (6–7 and 8–9), for which the period of fastest FOI reduction was predicted to be just before or during the first three surveys. Thus, although surveys 1 to 7 suggest a rather slow reduction of VT carriage for the younger age groups during the observational study, this seems to have been preceded by a period of high, short-term impact on VT carriage for those age groups (seen in the initial dynamics of Fig. 3a, b). Indeed, vaccine impact (reduction in VT carriage) at the time of the first survey was estimated to be 46.9% (95% CI 43.2–49.42) for the aggregated age group 0–5 years old. At the same time, the fastest reduction in FOI for the older age groups was predicted by the model to take place just before and during the first surveys, the time period in which survey data presents the largest reductions in VT carriage for those age groups (Fig. 1d). Overall, projected FOI dynamics suggest that PCV13 impact has been non-linear in time within age groups, with predicted periods of faster reductions in VT carriage being experienced by different ages in a sequential manner, from younger to older individuals.

### Sensitivity of vaccine impact based on transmission setting

**Fig. 5:**
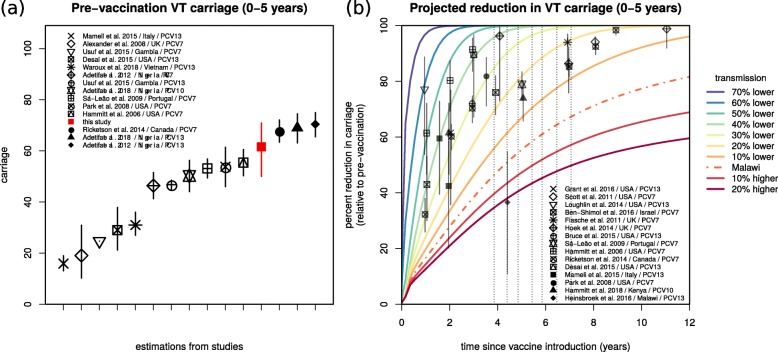
Estimated vaccine-type carriage and sensitivity of projections to baseline transmission in the context of other studies. **a** Estimated pre-vaccination vaccine-type carriage (and 95% CI) for the age group 0–5 years of age (red) in the context of carriage levels reported in other studies (in legend, Additional file 1: Table S6). **b** The baseline transmission coefficient (*β*) is varied by considering the 70%, 60%, 50%, 40%, 30%, 20% and 10% lower and 10% and 20% higher transmission than the estimated for Blantyre (Malawi, *β*_Malawi_) when fitting the observational study (e.g. 10% lower is 0.9*β_Malawi_). The impact projections for the age group 0–5 years old using the *β* estimated for Blantyre (Malawi) are presented by the dashed line (as in Fig. 3b). For visual purposes only, the means are shown, obtained from simulations sampling 100,000 parameter values from posteriors. The symbols and whiskers are measures of reported impact (carriage reduction) and 95% CIs for several published studies (in legend, Additional file 1: Table S5). The grey arrows mark the year of PCV13 introduction and the years of the four surveys

The projected impacts of Figs. 3 and 4 were based on the estimated transmission coefficients for Blantyre (Figs. 1b and 2d). To contextualise this particular transmission setting, we searched the literature for pre-vaccination VT carriage levels in other countries (Additional file 1: Table S6). The reported age groups were highly variable, and we therefore focused on the 0–5-year-old group for which more data points were available from a range of countries in North America, Africa, Europe and South-east Asia (Fig. 5a). Reported VT carriage in this age group was highly variable both between and within countries, with our estimation for Blantyre being on the higher end (61.58%, 95% CI 50.0–70.9%).

We further searched the literature for post-vaccination VT carriage levels in other countries and again focused on the age group 0–5 years old for which more data points were available (Additional file 1: Table S5, points with whiskers in Fig. 5b). The projected impact for Blantyre according to our model (dashed line) was notably lower than observed for other countries. A Malawi data point reported in the context of the Karonga District (Northern Malawi) had the closest impact to our projections in Blantyre (Southern Malawi), 4 to 5 years after PCV13 introduction [19].

Given that our posterior of vaccine efficacy (individual-level protection against carriage, Fig. 2c) was close to estimations from other regions of the world, we hypothesised that both the higher pre- and post-PCV13 VT carriage levels in Blantyre were likely due to a higher local force of infection compared to other regions. To demonstrate this, we simulated a range of alternative transmission settings in Blantyre, by varying both the transmission coefficients (*β* and *θ*) between − 70 and + 120% of their estimated posteriors (full exercise in Additional file 1: Figure S3). This sensitivity exercise showed that lowering local transmission by approximately − 30% was sufficient for the model to approximate short- and long-term vaccine impact observed in several other countries (Fig. 5b). Other age groups, for which far less data points were available, presented similar patterns (Additional file 1: Figure S4).

## Discussion

Using a dynamic model, we have reproduced observed changes in pneumococcal VT carriage following the introduction of PCV13 in Blantyre, Malawi. Similar to other modelling frameworks, we have considered the accumulation of natural immunity with age and have also allowed for heterogeneous transmission potentials within and between age groups. Including these factors allowed us to identify age-related characteristics of the local force of infection as the main determinants of post-PCV13 VT carriage dynamics in Blantyre.

A main motivation for developing our dynamic model was to explain the high residual VT carriage levels 7 years post-PCV13 introduction [22]. Studies from Kenya, The Gambia and South Africa have reported similar trends, with VT carriage remaining higher than in industrialised countries at similar post-vaccination time points. Compared to studies from other geographical regions, pre- and post-vaccination VT carriage in Blantyre was at the upper end of reported values across many countries (Fig. 5 and Additional file 1: Tables S5 and S6). Given that our estimate of vaccine efficacy (individual-level protection against carriage) was similar to reports from elsewhere (Fig. 2c, Additional file 1: Table S2), we tested the hypothesis that the observed and projected lower vaccine impact was likely a result of a higher force of infection in Blantyre compared to other regions. This force of infection was found to be characterised by different transmission potentials within and between age groups and particularly dominated by individuals younger than 5 years. Reflecting a variety of approaches and assumptions that can be found in other models [8, 11, 28], our framework is not able to discern if this assortative relationship with age is due to age-specific contact type patterns or susceptibility to colonisation. Nonetheless, our results strongly argue for the need for further characterisation of local contact, risk and transmission-route profiles (e.g. [45]), if we are to understand the myriad of reported PCV impacts across different demographic, social and epidemiological settings.

There were also the observations of vaccine impact (reduction in VT carriage) in unvaccinated age groups, and a particularly slow impact in younger vaccinated age groups during the surveys (Fig. 1d). The dynamic model helped explain these age-related responses, by showing that age groups have experienced periods of higher vaccine impact at different time points, sequentially, from younger to older groups. A major implication is that reduction in VT carriage in vaccinated younger age groups has been fastest between PCV13 introduction and 2015, when no carriage data was collected in Blantyre (but consistent with data collected in rural northern Malawi [19]). Thus, similarly to the conclusions of another modelling study [28], our results advocate for the essential role of dynamic models to understand post-PCV13 VT carriage, by critically accounting for local non-linear effects of pneumococcal transmission and vaccination, which may have significant implications for data interpretation.

Critical for low- and middle-income countries, as well as global initiatives such as Gavi [54], is that the impact of PCVs on pneumococcal VT carriage needs to be further improved if we are to maximise disease reduction. For high-burden countries like Malawi, in which post-PCV VT carriage data suggests that local epidemiological factors may dictate lower vaccine impact on carriage than elsewhere, region-specific improved vaccination schedules [19, 22] and catch-up campaigns [28] could help speed-up VT carriage reduction, improve herd protection and maximise cost-effectiveness. For this to be possible, we need to better understand local transmission profiles across ages, which are likely dictated by demographic and socio-economic factors, and strongly determine short- and long-term PCV impact.

In fact, participant socio-demographic data collected during the surveys has highlighted a generalised poor setting, with a large proportion of children (18 weeks to 7 years) living in houses with low infrastructure standards, high crowding and low possessions indices, and relying on shared communal water sources [22]. Although our modelling approach did not take into account such factors explicitly, they are known to favour transmission of infectious agents and could help explain our results of a high, local force of infection in Blantyre. Apart from the potential to tailor vaccine-related initiatives to local settings, more classic initiatives related to improving life-standards should also be taken into account when trying to maximise PCV impact and cost-effectiveness.

## Limitations

Data suggest that immune responses to PCV vaccines wane over time [22, 34]. In a meta-analysis study, PCV7 efficacy was estimated at 62% (CI 95% 52–72%) at 4 months post-vaccination, decreasing to 57% (CI 95% 50–65%) at 6 months, but remaining 42% (CI 95% 19–54%) at 5 years post-vaccination [34]. Models implicitly parametrising for duration of vaccine-induced protection (dVP) have typically followed a prior with minimum mean duration of 6 years [8, 11, 28, 34], but in one study dVP was estimated as 8.3 years (95% CI 5–20) [8]. Our framework does not explicitly include dVP, and this should be a line of future modelling research. Due to the time ranges studied for Blantyre (data were collected up to 7 years post-PCV13 introduction and projections made only up to the first 10 years), we argue that our results should be robust and only weakly influenced by not considering dVP. In light of the possibility that dVP is shorter than previously reported [22], our projections of vaccine impact should be seen as a best-case scenario; i.e. real long-term vaccine impact in Blantyre would likely be lower than projected by our model. Our framework also does not include niche competition between VT and non-VT pneumococci [11, 28, 34]. It is difficult to assert the impact of such competition in our main results, but it is unlikely that our conclusions would be significantly affected, since they are mostly based on factors which have not been reported to be associated with type competition directly (e.g. age-specific transmission). We demonstrated the importance of age-related heterogeneities in the transmission matrix but were unable to disentangle the effects of contact type and frequency versus susceptibility and transmissibility. This limitation was by design as we avoided increasing model complexity, but is a topic of future modelling research as we gather carriage data covering longer time periods into the post-PCV era. Finally, it is reasonable to assume that the vaccine could impact duration of carriage (but see [55]) as a consequence of changes in the accumulation of immunity through reduced natural exposure. We have not explored this in our current study since the explicit inclusion of such mechanism would require the addition of multiple parameters for which insufficient information is currently unavailable.

## Conclusion

In Blantyre, vaccine efficacy (individual-level protection against carriage) across ages and time was estimated at 66.87% (95% CI 50.49–82.26%), similar to reports from other countries. However, local transmission potential in Blantyre is likely to be higher than in other countries and also heterogeneous among age groups, with a particular contribution from younger children. While PCV13 is achieving positive outcomes in Blantyre [19, 56], a local higher and age-dependent force of infection is dictating a lower long-term vaccine impact (population-level carriage reduction) than reported elsewhere. Finally, the combination of age-related transmission heterogeneities and routinely vaccinating infants has led to non-linear responses in terms of vaccine impact across ages and time, with general implications on post-vaccination VT carriage data interpretation. Together, these findings suggest that in regions with lower than desired PCV impact on VT carriage, alternative vaccine schedules and catch-up campaigns targeting children < 5 years of age should be further evaluated.

## Supplementary information

**Additional file 1.** Methodological details, literature support and complimentary results.

## Data Availability

Carriage data used in this study is made available in Swarthout et al. [22], where the carriage study is described in detail.

## Abbreviations

bMCMC: Bayesian Markov chain Monte Carlo
CI: Confidence interval
dVP: Duration of vaccine-induced protection
FOI: Force of infection
NVT: Non-vaccine type
ODE: Ordinary-differential equations
PCV: Pneumococcal conjugate vaccine
VT: Vaccine type

## References

[1] Brown J, Hammerschmidt S, Orihuela C, editors. *Streptococcus Pneumoniae*: molecular mechanisms of host-pathogen interactions. 1st ed: Elsevier; 2015. 10.1016/C2012-0-00722-3.

[2] Levine OS, O’Brien KL, Knoll M (2006). Pneumococcal vaccination in developing countries. Lancet.

[3] Simell B, Auranen K, Käyhty H, Goldblatt D, Dagan R, O’Brien KL (2012). The fundamental link between pneumococcal carriage and disease. Expert Rev Vaccines.

[4] Weinberger DM, Malley R, Lipsitch M (2011). Serotype replacement in disease after pneumococcal vaccination. Lancet.

[5] Watkins ER, Penman BS, Lourenço J (2015). Vaccination drives changes in metabolic and virulence profiles of Streptococcus pneumoniae. PLoS Pathog.

[6] Lourenço J, Wikramaratna PSPS, Gupta S (2015). MANTIS: an R package that simulates multilocus models of pathogen evolution. BMC Bioinformatics.

[7] Ashby Ben, Watkins Eleanor, Lourenço José, Gupta Sunetra, Foster Kevin R. (2017). Competing species leave many potential niches unfilled. Nature Ecology & Evolution.

[8] Melegaro A, Choi YH, George R, Edmunds WJ, Miller E, Gay NJ (2010). Dynamic models of pneumococcal carriage and the impact of the heptavalent pneumococcal conjugate vaccine on invasive pneumococcal disease. BMC Infect Dis.

[9] Bottomley C, Roca A, Hill PC, Greenwood B, Isham V (2013). A mathematical model of serotype replacement in pneumococcal carriage following vaccination. J R Soc Interface.

[10] Adetifa Ifedayo M. O., Antonio Martin, Okoromah Christy A. N., Ebruke Chinelo, Inem Victor, Nsekpong David, Bojang Abdoulie, Adegbola Richard A. (2012). Pre-Vaccination Nasopharyngeal Pneumococcal Carriage in a Nigerian Population: Epidemiology and Population Biology. PLoS ONE.

[11] Le Polain de Waroux O, Edmunds WJ, Takahashi K (2018). Predicting the impact of pneumococcal conjugate vaccine programme options in Vietnam. Hum Vaccin Immunother.

[12] Cohen R, Levy C, Bonnet E (2010). Dynamic of pneumococcal nasopharyngeal carriage in children with acute otitis media following PCV7 introduction in France. Vaccine.

[13] Collins DA, Hoskins A, Snelling T (2017). Predictors of pneumococcal carriage and the effect of the 13-valent pneumococcal conjugate vaccination in the Western Australian aboriginal population. Pneumonia.

[14] Spijkerman J, van Gils EJM, Veenhoven RH (2011). Carriage of *Streptococcus pneumoniae* 3 years after start of vaccination program, the Netherlands. Emerg Infect Dis.

[15] Desai AP, Sharma D, Crispell EK (2015). Decline in pneumococcal nasopharyngeal carriage of vaccine serotypes after the introduction of the 13-valent pneumococcal conjugate vaccine in children in Atlanta, Georgia. Pediatr Infect Dis J.

[16] Bruce MG, Singleton R, Bulkow L (2015). Impact of the 13-valent pneumococcal conjugate vaccine (pcv13) on invasive pneumococcal disease and carriage in Alaska. Vaccine.

[17] Hammitt LL, Akech DO, Morpeth SC (2014). Population effect of 10-valent pneumococcal conjugate vaccine on nasopharyngeal carriage of Streptococcus pneumoniae and non-typeable Haemophilus influenzae in Kilifi, Kenya: findings from cross-sectional carriage studies. Lancet Glob Heal.

[18] Hammitt LL, Etyang AO, Morpeth SC (2019). Effect of ten-valent pneumococcal conjugate vaccine on invasive pneumococcal disease and nasopharyngeal carriage in Kenya: a longitudinal surveillance study. Lancet.

[19] Heinsbroek Ellen, Tafatatha Terence, Phiri Amos, Swarthout Todd D, Alaerts Maaike, Crampin Amelia C, Chisambo Christina, Mwiba Oddie, Read Jonathan M, French Neil (2018). Pneumococcal carriage in households in Karonga District, Malawi, before and after introduction of 13-valent pneumococcal conjugate vaccination. Vaccine.

[20] Roca A, Bojang A, Bottomley C (2015). Effect on nasopharyngeal pneumococcal carriage of replacing PCV7 with PCV13 in the expanded programme of immunization in the Gambia. Vaccine.

[21] Nunes MC, Jones SA, Groome MJ (2015). Acquisition of Streptococcus pneumoniae in south African children vaccinated with 7-valent pneumococcal conjugate vaccine at 6, 14 and 40 weeks of age. Vaccine.

[22] Swarthout TD, Fronterre C, Lourenço J, et al. High residual prevalence of vaccine-serotype *Streptococcus pneumoniae* carriage after introduction of a pneumococcal conjugate vaccine in Malawi: a prospective serial cross-sectional study. bioRxiv. 2019; https://www.biorxiv.org/content/10.1101/445999v2.

[23] Obolski U, Lourenço J, Thompson C, Thompson R, Gori A, Gupta S (2018). Vaccination can drive an increase in frequencies of antibiotic resistance among nonvaccine serotypes of Streptococcus pneumoniae. Proc Natl Acad Sci.

[24] McCormick AW, Whitney CG, Farley MM (2003). Geographic diversity and temporal trends of antimicrobial resistance in Streptococcus pneumoniae in the United States. Nat Med.

[25] Lehtinen S, Blanquart F, Croucher NJ, Turner P, Lipsitch M, Fraser C (2017). Evolution of antibiotic resistance is linked to any genetic mechanism affecting bacterial duration of carriage. Proc Natl Acad Sci.

[26] Huang SS, Finkelstein JA, Lipsitch M (2005). Modeling community- and individual-level effects of child-care center attendance on pneumococcal carriage. Clin Infect Dis.

[27] Van Effelterre T, Moore MR, Fierens F (2010). A dynamic model of pneumococcal infection in the United States: implications for prevention through vaccination. Vaccine.

[28] Flasche S, Ojal J, Le Polain de Waroux O (2017). Assessing the efficiency of catch-up campaigns for the introduction of pneumococcal conjugate vaccine: a modelling study based on data from PCV10 introduction in Kilifi, Kenya. BMC Med.

[29] Melegaro A, Choi Y, Pebody R, Gay N (2007). Pneumococcal carriage in United Kingdom families: estimating serotype-specific transmission parameters from longitudinal data. Am J Epidemiol.

[30] Melegaro A, Gay NJ, Medley GF (2004). Estimating the transmission parameters of pneumococcal carriage in households. Epidemiol Infect.

[31] Nurhonen Markku, Cheng Allen C., Auranen Kari (2013). Pneumococcal Transmission and Disease In Silico: A Microsimulation Model of the Indirect Effects of Vaccination. PLoS ONE.

[32] Auranen K, Mehtälä J, Tanskanen A, Kaltoft MS (2010). Between-strain competition in acquisition and clearance of pneumococcal carriage epidemiologic evidence from a longitudinal study of day-care children. Am J Epidemiol.

[33] Erästö P, Hoti F, Granat SM, Mia Z, Mäkelä PH, Auranen K (2010). Modelling multi-type transmission of pneumococcal carriage in Bangladeshi families. Epidemiol Infect.

[34] Le Polain De Waroux O, Flasche S, Prieto-Merino D, Goldblatt D, Edmunds WJ (2015). The efficacy and duration of protection of pneumococcal conjugate vaccines against nasopharyngeal carriage: a meta-regression model. Pediatr Infect Dis J.

[35] Ojal J, Griffiths U, Hammitt LL (2019). Sustaining pneumococcal vaccination after transitioning from Gavi support: a modelling and cost-effectiveness study in Kenya. Lancet Glob Heal.

[36] Satzke C, Turner P, Virolainen-Julkunen A (2013). Standard method for detecting upper respiratory carriage of Streptococcus pneumoniae: updated recommendations from the World Health Organization pneumococcal carriage working group. Vaccine.

[37] Lourenço J, de Lima MM, Faria NR, et al. Epidemiological and ecological determinants of Zika virus transmission in an urban setting. Elife. 2017;6. 10.7554/eLife.29820. 10.7554/eLife.29820 PMC5638629 28887877

[38] Faria NR, da Costa AC, Lourenço J (2017). Genomic and epidemiological characterisation of a dengue virus outbreak among blood donors in Brazil. Sci Rep.

[39] McNaughton AL, Lourenço J, Hattingh L (2019). HBV vaccination and PMTCT as elimination tools in the presence of HIV: insights from a clinical cohort and dynamic model. BMC Med.

[40] Weiser JN, Ferreira DM, Paton JC (2018). Streptococcus pneumoniae: transmission, colonization and invasion. Nat Rev Microbiol.

[41] Hogberg L, Geli P, Ringberg H, Melander E, Lipsitch M, Ekdahl K (2007). Age- and serogroup-related differences in observed durations of nasopharyngeal carriage of penicillin-resistant pneumococci. J Clin Microbiol.

[42] Vehtari A, Gelman A, Gabry J (2016). Practical Bayesian model evaluation using leave-one-out cross-validation and WAIC. Stat Comput.

[43] Vehtari A, Gabry J, Yao Y, Gelman A (2018). loo: Efficient leave-one-out cross-validation and WAIC for Bayesian models.

[44] Yao Y, Vehtari A, Simpson D, Gelman A. Using stacking to average Bayesian predictive distributions. Bayesian Anal. 2018;13(3):917–1003.

[45] le Polain de Waroux O, Cohuet S, Ndazima D (2018). Characteristics of human encounters and social mixing patterns relevant to infectious diseases spread by close contact: a survey in Southwest Uganda. BMC Infect Dis.

[46] Althouse BM, Hammitt LL, Grant L (2017). Identifying transmission routes of Streptococcus pneumoniae and sources of acquisitions in high transmission communities. Epidemiol Infect.

[47] Ojal J, Flasche S, Hammitt LL (2017). Sustained reduction in vaccine-type invasive pneumococcal disease despite waning effects of a catch-up campaign in Kilifi, Kenya: a mathematical model based on pre-vaccination data. Vaccine.

[48] Camilli R, Daprai L, Cavrini F (2013). Pneumococcal carriage in young children one year after introduction of the 13-Valent conjugate vaccine in Italy. PLoS One.

[49] Mossong J, Hens N, Jit M (2008). Social contacts and mixing patterns relevant to the spread of infectious diseases. PLoS Med.

[50] Kiti Moses Chapa, Kinyanjui Timothy Muiruri, Koech Dorothy Chelagat, Munywoki Patrick Kiio, Medley Graham Francis, Nokes David James (2014). Quantifying Age-Related Rates of Social Contact Using Diaries in a Rural Coastal Population of Kenya. PLoS ONE.

[51] Van Hoek AJ, Sheppard CL, Andrews NJ (2014). Pneumococcal carriage in children and adults two years after introduction of the thirteen valent pneumococcal conjugate vaccine in England. Vaccine.

[52] Mameli C, Fabiano V, Daprai L (2015). A longitudinal study of streptococcus pneumoniae carriage in healthy children in the 13-valent pneumococcal conjugate vaccine era. Hum Vaccin Immunother.

[53] Sá-Leão R, Nunes S, Brito-Avô A (2009). Changes in pneumococcal serotypes and antibiotypes carried by vaccinated and unvaccinated day-care centre attendees in Portugal, a country with widespread use of the seven-valent pneumococcal conjugate vaccine. Clin Microbiol Infect.

[54] Gavi: the vaccine alliance. https://www.gavi.org. Accessed 28 Aug 2019.

[55] Ferreira DM, Jambo KC, Gordon SB (2011). Experimental human pneumococcal carriage models for vaccine research. Trends Microbiol.

[56] McCollum ED, Nambiar B, Deula R (2017). Impact of the 13-valent pneumococcal conjugate vaccine on clinical and hypoxemic childhood pneumonia over three years in Central Malawi: an observational study. PLoS One.

