# Supplementary material for: Determinants of high residual post-PCV13 pneumococcal vaccine-type carriage in Blantyre, Malawi: a modelling study
Source: BMC Med. 2019 Dec 5;17:219. doi: 10.1186/s12916-019-1450-2 (PMC6894346; doi:10.1186/s12916-019-1450-2)
Supplement: Supplementary file 1 — Additional file 1. Methodological details, literature support and complimentary results. [file 12916_2019_1450_MOESM1_ESM.pdf]

Determinants of high residual post-PCV13 pneumococcal vaccine type carriage in Blantyre, Malawi: a modelling study.

Additional File 1: methodological details, literature support and complimentary results.

Table of Contents

Ordinary-differential equations (ODE)........1
Definition of birth rate for a constant population size........2
Expressions for forces of infection........2
Expressions for model carriage levels........3
Markov-chain Monte-Carlo fitting approach........4
Model age-structure at equilibrium........4
Literature support for estimated durations of carriage........5
Literature support for estimated vaccine efficacy against carriage........5
Literature support and model sensitivity to different transmission matrices........6
Literature review of carriage reduction in time........9
Literature review of pre-vaccination carriage levels........10
Extra results for sensitivity of vaccine impact projections........11
Observational study data........12
References........13

Ordinary-differential equations (ODE)

Equations for the vaccinated (equations 1-14) and unvaccinated (equations 15-28) age-groups, with model diagram in Figure 1 of main text:

$$
\begin{aligned}
dS_0/d_t &= b(1-\rho) - \lambda_0 S_0 + \gamma_{0-2} C_0 - a_0 S_0 - \mu_0 S_0 && (1)\\
dC_0/d_t &= \lambda_0 S_0 - \gamma_{0-2} C_0 - a_0 C_0 - \mu_0 C_0 && (2)\\
dS_1/d_t &= a_0 S_0 - \lambda_1 S_1 + \gamma_{0-2} C_1 - a_1 S_1 - \mu_1 S_1 && (3)\\
dC_1/d_t &= a_0 C_0 + \lambda_1 S_1 - \gamma_{0-2} C_1 - a_1 C_1 - \mu_1 C_1 && (4)\\
dS_2/d_t &= a_1 S_1 - \lambda_2 S_2 + \gamma_{0-2} C_2 - a_2 S_2 - \mu_2 S_2 && (5)\\
dC_2/d_t &= a_1 C_1 + \lambda_2 S_2 - \gamma_{0-2} C_2 - a_2 C_2 - \mu_2 C_2 && (6)\\
dS_{3-5}/d_t &= a_2 S_2 - \lambda_{3-5} S_{3-5} + \gamma_{3-5} C_{3-5} - a_{3-5} S_{3-5} - \mu_{3-5} S_{3-5} && (7)\\
dC_{3-5}/d_t &= a_2 C_2 + \lambda_{3-5} S_{3-5} - \gamma_{3-5} C_{3-5} - a_{3-5} C_{3-5} - \mu_{3-5} C_{3-5} && (8)\\
dS_{6-7}/d_t &= a_{3-5} S_{3-5} - \lambda_{6-7} S_{6-7} + \gamma_{6-7} C_{6-7} - a_{6-7} S_{6-7} - \mu_{6-7} S_{6-7} && (9)\\
dC_{6-7}/d_t &= a_{3-5} C_{3-5} + \lambda_{6-7} S_{6-7} - \gamma_{6-7} C_{6-7} - a_{6-7} C_{6-7} - \mu_{6-7} C_{6-7} && (10)\\
dS_{8-9}/d_t &= a_{6-7} S_{6-7} - \lambda_{8-9} S_{8-9} + \gamma_{8+} C_{8-9} - a_{8-9} S_{8-9} - \mu_{8-9} S_{8-9} && (11)\\
dC_{8-9}/d_t &= a_{6-7} C_{6-7} + \lambda_{8-9} S_{8-9} - \gamma_{8+} C_{8-9} - a_{8-9} C_{8-9} - \mu_{8-9} C_{8-9} && (12)\\
dS_{10+}/d_t &= a_{8-9} S_{8-9} - \lambda_{10+} S_{10+} + \gamma_{8+} C_{10+} - \mu_{10+} S_{10+} && (13)\\
dC_{10+}/d_t &= a_{8-9} C_{8-9} + \lambda_{10+} S_{10+} - \gamma_{8+} C_{10+} - \mu_{10+} C_{10+} && (14)
\end{aligned}
$$

$$
\begin{aligned}
dS_0^v/d_t &= b\rho - (1-\zeta)\lambda_0 S_0^v + \gamma_{0-2} C_0^v - a_0 S_0^v - \mu_0 S_0^v & (15)\\
dC_0^v/d_t &= (1-\zeta)\lambda_0 S_0^v - \gamma_{0-2} C_0^v - a_0 C_0^v - \mu_0 C_0^v & (16)\\
dS_1^v/d_t &= a_0 S_0^v - (1-\zeta)\lambda_1 S_1^v + \gamma_{0-2} C_1^v - a_1 S_1^v - \mu_1 S_1^v & (17)\\
dC_1^v/d_t &= a_0 C_0^v + (1-\zeta)\lambda_1 S_1^v - \gamma_{0-2} C_1^v - a_1 C_1^v - \mu_1 C_1^v & (18)\\
dS_2^v/d_t &= a_1 S_1^v - (1-\zeta)\lambda_2 S_2^v + \gamma_{0-2} C_2^v - a_2 S_2^v - \mu_2 S_2^v & (19)\\
dC_2^v/d_t &= a_1 C_1^v + (1-\zeta)\lambda_2 S_2^v - \gamma_{0-2} C_2^v - a_2 C_2^v - \mu_2 C_2^v & (20)\\
dS_{3-5}^v/d_t &= a_2 S_2^v - (1-\zeta)\lambda_{3-5} S_{3-5}^v + \gamma_{3-5} C_{3-5}^v - a_{3-5} S_{3-5}^v - \mu_{3-5} S_{3-5}^v & (21)\\
dC_{3-5}^v/d_t &= a_2 C_2^v + (1-\zeta)\lambda_{3-5} S_{3-5}^v - \gamma_{3-5} C_{3-5}^v - a_{3-5} C_{3-5}^v - \mu_{3-5} C_{3-5}^v & (22)\\
dS_{6-7}^v/d_t &= a_{3-5} S_{3-5}^v - (1-\zeta)\lambda_{6-7} S_{6-7}^v + \gamma_{6-7} C_{6-7}^v - a_{6-7} S_{6-7}^v - \mu_{6-7} S_{6-7}^v & (23)\\
dC_{6-7}^v/d_t &= a_{3-5} C_{3-5}^v + (1-\zeta)\lambda_{6-7} S_{6-7}^v - \gamma_{6-7} C_{6-7}^v - a_{6-7} C_{6-7}^v - \mu_{6-7} C_{6-7}^v & (24)\\
dS_{8-9}^v/d_t &= a_{6-7} S_{6-7}^v - (1-\zeta)\lambda_{8-9} S_{8-9}^v + \gamma_{8+} C_{8-9}^v - a_{8-9} S_{8-9}^v - \mu_{8-9} S_{8-9}^v & (25)\\
dC_{8-9}^v/d_t &= a_{6-7} C_{6-7}^v + (1-\zeta)\lambda_{8-9} S_{8-9}^v - \gamma_{8+} C_{8-9}^v - a_{8-9} C_{8-9}^v - \mu_{8-9} C_{8-9}^v & (26)\\
dS_{10+}^v/d_t &= a_{8-9} S_{8-9}^v - (1-\zeta)\lambda_{10+} S_{10+}^v + \gamma_{8+} C_{10+}^v - \mu_{10+} S_{10+}^v & (27)\\
dC_{10+}^v/d_t &= a_{8-9} C_{8-9}^v + (1-\zeta)\lambda_{10+} S_{10+}^v - \gamma_{8+} C_{10+}^v - \mu_{10+} C_{10+}^v & (28)
\end{aligned}
$$

Definition of birth rate for a constant population size

Given that the total population size is kept constant, the birth rate is a composite expression of deaths across ages:

$$
\begin{aligned}
b = \; & \mu_0(S_0 + C_0 + S_0^v + C_0^v) + \\
& \mu_1(S1 + C1 + S_1^v + C_1^v) + \\
& \mu_2(S2 + C2 + S_2^v + C_2^v) + \\
& \mu_{3-5}(S_{3-5} + C_{3-5} + S_{3-5}^v + C_{3-5}^v) + \\
& \mu_{6-7}(S_{6-7} + C_{6-7} + S_{6-7}^v + C_{6-7}^v) + \\
& \mu_{8-9}(S_{8-9} + C_{8-9} + S_{8-9}^v + C_{8-9}^v) + \\
& \mu_{10+}(S_{10+} + C_{10+} + S_{10+}^v + C_{10+}^v) \qquad (29)
\end{aligned}
$$

Expressions for forces of infection

The forces of infection of the different model variants (see Figure S1) are defined as follows.

model 0-5 years + Assortative

$$
\begin{aligned}
\lambda_0 &= \theta Ca_0 + \theta Ca_1 + \theta Ca_2 + \theta Ca_{3-5} + \beta Ca_{6-7} + \beta Ca_{8-9} + \beta Ca_{10+} & (30)\\
\lambda_1 &= \theta Ca_0 + \theta Ca_1 + \theta Ca_2 + \theta Ca_{3-5} + \beta Ca_{6-7} + \beta Ca_{8-9} + \beta Ca_{10+} & (31)\\
\lambda_2 &= \theta Ca_0 + \theta Ca_1 + \theta Ca_2 + \theta Ca_{3-5} + \beta Ca_{6-7} + \beta Ca_{8-9} + \beta Ca_{10+} & (32)\\
\lambda_{3-5} &= \theta Ca_0 + \theta Ca_1 + \theta Ca_2 + \theta Ca_{3-5} + \beta Ca_{6-7} + \beta Ca_{8-9} + \beta Ca_{10+} & (33)\\
\lambda_{6-7} &= \beta Ca_0 + \beta Ca_1 + \beta Ca_2 + \beta Ca_{3-5} + \theta Ca_{6-7} + \beta Ca_{8-9} + \beta Ca_{10+} & (34)\\
\lambda_{8-9} &= \beta Ca_0 + \beta Ca_1 + \beta Ca_2 + \beta Ca_{3-5} + \beta Ca_{6-7} + \theta Ca_{8-9} + \beta Ca_{10+} & (35)\\
\lambda_{10+} &= \beta Ca_0 + \beta Ca_1 + \beta Ca_2 + \beta Ca_{3-5} + \beta Ca_{6-7} + \beta Ca_{8-9} + \beta Ca_{10+} & (36)
\end{aligned}
$$

model 1-5 years + Assortative

$$
\begin{aligned}
\lambda_0 &= \beta Ca_0 + \beta Ca_1 + \beta Ca_2 + \beta Ca_{3-5} + \beta Ca_{6-7} + \beta Ca_{8-9} + \beta Ca_{10+} && (37)\\
\lambda_1 &= \beta Ca_0 + \theta Ca_1 + \theta Ca_2 + \theta Ca_{3-5} + \beta Ca_{6-7} + \beta Ca_{8-9} + \beta Ca_{10+} && (38)\\
\lambda_2 &= \beta Ca_0 + \theta Ca_1 + \theta Ca_2 + \theta Ca_{3-5} + \beta Ca_{6-7} + \beta Ca_{8-9} + \beta Ca_{10+} && (39)\\
\lambda_{3-5} &= \beta Ca_0 + \theta Ca_1 + \theta Ca_2 + \theta Ca_{3-5} + \beta Ca_{6-7} + \beta Ca_{8-9} + \beta Ca_{10+} && (40)\\
\lambda_{6-7} &= \beta Ca_0 + \beta Ca_1 + \beta Ca_2 + \beta Ca_{3-5} + \theta Ca_{6-7} + \beta Ca_{8-9} + \beta Ca_{10+} && (41)\\
\lambda_{8-9} &= \beta Ca_0 + \beta Ca_1 + \beta Ca_2 + \beta Ca_{3-5} + \beta Ca_{6-7} + \theta Ca_{8-9} + \beta Ca_{10+} && (42)\\
\lambda_{10+} &= \beta Ca_0 + \beta Ca_1 + \beta Ca_2 + \beta Ca_{3-5} + \beta Ca_{6-7} + \beta Ca_{8-9} + \beta Ca_{10+} && (43)
\end{aligned}
$$

model 2-5 years + Assortative

$$
\begin{aligned}
\lambda_0 &= \beta Ca_0 + \beta Ca_1 + \beta Ca_2 + \beta Ca_{3-5} + \beta Ca_{6-7} + \beta Ca_{8-9} + \beta Ca_{10+} && (44)\\
\lambda_1 &= \beta Ca_0 + \beta Ca_1 + \beta Ca_2 + \beta Ca_{3-5} + \beta Ca_{6-7} + \beta Ca_{8-9} + \beta Ca_{10+} && (45)\\
\lambda_2 &= \beta Ca_0 + \beta Ca_1 + \theta Ca_2 + \theta Ca_{3-5} + \beta Ca_{6-7} + \beta Ca_{8-9} + \beta Ca_{10+} && (46)\\
\lambda_{3-5} &= \beta Ca_0 + \beta Ca_1 + \theta Ca_2 + \theta Ca_{3-5} + \beta Ca_{6-7} + \beta Ca_{8-9} + \beta Ca_{10+} && (47)\\
\lambda_{6-7} &= \beta Ca_0 + \beta Ca_1 + \beta Ca_2 + \beta Ca_{3-5} + \theta Ca_{6-7} + \beta Ca_{8-9} + \beta Ca_{10+} && (48)\\
\lambda_{8-9} &= \beta Ca_0 + \beta Ca_1 + \beta Ca_2 + \beta Ca_{3-5} + \beta Ca_{6-7} + \theta Ca_{8-9} + \beta Ca_{10+} && (49)\\
\lambda_{10+} &= \beta Ca_0 + \beta Ca_1 + \beta Ca_2 + \beta Ca_{3-5} + \beta Ca_{6-7} + \beta Ca_{8-9} + \beta Ca_{10+} && (50)
\end{aligned}
$$

model Assortative

$$
\begin{aligned}
\lambda_0 &= \theta Ca_0 + \beta Ca_1 + \beta Ca_2 + \beta Ca_{3-5} + \beta Ca_{6-7} + \beta Ca_{8-9} + \beta Ca_{10+} && (51)\\
\lambda_1 &= \beta Ca_0 + \theta Ca_1 + \beta Ca_2 + \beta Ca_{3-5} + \beta Ca_{6-7} + \beta Ca_{8-9} + \beta Ca_{10+} && (52)\\
\lambda_2 &= \beta Ca_0 + \beta Ca_1 + \theta Ca_2 + \beta Ca_{3-5} + \beta Ca_{6-7} + \beta Ca_{8-9} + \beta Ca_{10+} && (53)\\
\lambda_{3-5} &= \beta Ca_0 + \beta Ca_1 + \beta Ca_2 + \theta Ca_{3-5} + \beta Ca_{6-7} + \beta Ca_{8-9} + \beta Ca_{10+} && (54)\\
\lambda_{6-7} &= \beta Ca_0 + \beta Ca_1 + \beta Ca_2 + \beta Ca_{3-5} + \theta Ca_{6-7} + \beta Ca_{8-9} + \beta Ca_{10+} && (55)\\
\lambda_{8-9} &= \beta Ca_0 + \beta Ca_1 + \beta Ca_2 + \beta Ca_{3-5} + \beta Ca_{6-7} + \theta Ca_{8-9} + \beta Ca_{10+} && (56)\\
\lambda_{10+} &= \beta Ca_0 + \beta Ca_1 + \beta Ca_2 + \beta Ca_{3-5} + \beta Ca_{6-7} + \beta Ca_{8-9} + \beta Ca_{10+} && (57)
\end{aligned}
$$

model Homogeneous

$$
\begin{aligned}
\lambda_0 &= \beta Ca_0 + \beta Ca_1 + \beta Ca_2 + \beta Ca_{3-5} + \beta Ca_{6-7} + \beta Ca_{8-9} + \beta Ca_{10+} && (58)\\
\lambda_1 &= \beta Ca_0 + \beta Ca_1 + \beta Ca_2 + \beta Ca_{3-5} + \beta Ca_{6-7} + \beta Ca_{8-9} + \beta Ca_{10+} && (59)\\
\lambda_2 &= \beta Ca_0 + \beta Ca_1 + \beta Ca_2 + \beta Ca_{3-5} + \beta Ca_{6-7} + \beta Ca_{8-9} + \beta Ca_{10+} && (60)\\
\lambda_{3-5} &= \beta Ca_0 + \beta Ca_1 + \beta Ca_2 + \beta Ca_{3-5} + \beta Ca_{6-7} + \beta Ca_{8-9} + \beta Ca_{10+} && (61)\\
\lambda_{6-7} &= \beta Ca_0 + \beta Ca_1 + \beta Ca_2 + \beta Ca_{3-5} + \beta Ca_{6-7} + \beta Ca_{8-9} + \beta Ca_{10+} && (62)\\
\lambda_{8-9} &= \beta Ca_0 + \beta Ca_1 + \beta Ca_2 + \beta Ca_{3-5} + \beta Ca_{6-7} + \beta Ca_{8-9} + \beta Ca_{10+} && (63)\\
\lambda_{10+} &= \beta Ca_0 + \beta Ca_1 + \beta Ca_2 + \beta Ca_{3-5} + \beta Ca_{6-7} + \beta Ca_{8-9} + \beta Ca_{10+} && (64)
\end{aligned}
$$

Expressions for model carriage levels

Levels of carriage in the ODE model are calculated as the proportion of individuals within an age-group, with a particular vaccine status, that are carriers at the time of the surveys. For example:

$$
\begin{aligned}
Ca_0 &= C_0 + C_0^v && (65)\\
Ca_1 &= C_1 + C_1^v && (66)\\
Ca_2 &= C_2 + C_2^v && (67)\\
Ca_{3-5} &= C_{3-5} + C_{3-5}^v && (68)\\
Ca_{6-7} &= C_{6-7} + C_{6-7}^v && (69)\\
Ca_{8-9} &= C_{8-9} + C_{8-9}^v && (70)\\
Ca_{10+} &= C_{10+} + C_{10+}^v && (71)
\end{aligned}
$$

Markov-chain Monte-Carlo fitting approach

We used a Bayesian Markov chain Monte Carlo (bMCMC) approach, developed and used by us in other modelling studies [1]–[3]. The proposal distributions (*q*) of each parameter are defined as Gaussian (symmetric), effectively implementing a random walk Metropolis kernel. We define our acceptance probability α of a parameter set ϴ* as:

$$\alpha \;=\; min\{1, \frac{\pi(y|\Theta^{\star})p(\Theta^{\star})q(\Theta^{o}|\Theta^{\star})}{\pi(y|\Theta^{o})p(\Theta^{o})q(\Theta^{\star}|\Theta^{o})}\} \qquad (72)$$

where ϴ* and ϴo are the proposed and current (accepted) parameter sets (respectively); Π(y | ϴ*) and Π(y | ϴo) are the likelihoods of the observed data (y, Table S7) under the ODE output given the parameter sets; p(ϴo) and p(ϴ*) are the prior-related probabilities given each parameter set.

For simplicity and because all fitted variables are proportions, the likelihoods π were calculated as the product of conditional Gaussian probabilities (*fϴ(yi)*). The likelihood is the product the conditional probabilities of all variables, and can be formally expressed as:

$$\pi(y|\Theta) \;=\; \prod_{i=1}^{N}[f_{\theta}(y_i)] \qquad (73)$$

We used Gaussian probabilities instead of Binomial for mathematical and computational convenience. It should be noted, however, that the Binomial distribution is well approximated by the Gaussian distribution under the common rule of thumb of n*min(p,1-p)>5 (n= sample size, p=carriage level in an age-group), which is not violated in our observational dataset [4] .

Model age-structure at equilibrium

The age-groups used in the modelling framework were based on the groups for which carriage data was collected in the rolling prospective nasopharyngeal carriage surveys [4] . To confirm that ageing was robust in the ODE model, we compared the model's proportions of the population in each age-group (at equilibrium, before vaccination) to the known proportions for Malawi (country's age pyramid for the year of 2019 available at https://www.populationpyramid.net/malawi/2019/).

Because the age pyramid groups under 10 years of age were different from the model's, we first compared proportions below and above 10 years of age: we obtained mean model proportions of 0.150 and 0.849 versus age pyramid proportions of 0.158 and 0.842 (for <10 and >=10 years, respectively). We then attempted to approximate the age-grouping under 10 years of age; using the pyramid's age-groups 0-4 and 5-9 years. For this, we aggregated the model's proportions with all 0, 1, 2, and two-thirds of 3-5 years old, as well as aggregated the proportions with one-third of 3-5, and all 6-7 and 8-9 years old. The model's proportions were 0.078 and 0.072, compared to the pyramid's 0.084 and 0.074 (for 0-4 and 5-9 years, respectively). The proportions between the model and the age pyramid were very similar, thus validating the ageing implemented in the ODE model.

Literature support for estimated durations of carriage

While we base our priors on the study by Hogberg and colleagues [5], there is vast support in the literature for both the use of similar values and the key assumption of a decrease in duration of carriage with age. We summarize this support in Table S1 below.

Age-group	Duration	Country	Ref.
0-1 years	71	UK	[11]–[13]
2-4 years	28	UK	[11]–[13]
5-17 years	18	UK	[11]–[13]
18+ years	17	UK	[11]–[13]
0-1 years	30	Sweden	[14]
1-4 years	21	Sweden	[14]
5-6 years	13	Sweden	[14]
7-18 years	15	Sweden	[6]
18+ years	14	Sweden	[6]
0-2 years	60*	Thailand & Myanmar	[7]
Mothers	31*	Thailand & Myanmar	[7]
6-11 months	48.5*	Kenya	[8]
3-59 months	31.3*	Kenya	[8]
0-7 years	44*	Finland	[9]
7+ years	36	Finland	[9]
0-5 years	42.6*	Bangladesh	[10]
5+ years	38	Bangladesh	[10]

Table S1 – Support for decrease of duration of carriage with age. Mean values are presented and * marks values for which 95% CI are available in the original study. Grey and white backgrounds are a visual cue for different countries.

Literature support for estimated vaccine efficacy against carriage

The posterior for vaccine efficacy against carriage (ζ) was shown in the main text to be in the range of estimates obtained from other studies. Table S2 presents the values reported in such studies.

VE	Vaccine	Ref.	Country / Region
56% (95% CI 41 - 72)	PCV10	[15]	Kenya
66% (95% CI 38 - 82)	PCV10	[16]	Kenya
63% (95% CI 53 - 73)	Meta	[17]	Meta-analysis
78% (95% CI 64 - 92)	PCV13	[18]	Vietnam
59% (95% CI 49 - 68)	PCV10	[19]	Kenya

Table S2 – Support for estimated vaccine efficacy. Mean values, 95% CI, vaccine, reference and country are presented for each of the studies. These quantifications were reported in the original articles (were not estimated by us on reported data).

Literature support and model sensitivity to different transmission matrices

We formulated five different transmission matrices (Figure S1). One matrix assumed that transmission potential between age-groups is homogeneous (Figure S1e), while the other four assumed variations of inhomogeneous transmission potential between the groups (Figure S1a-d).

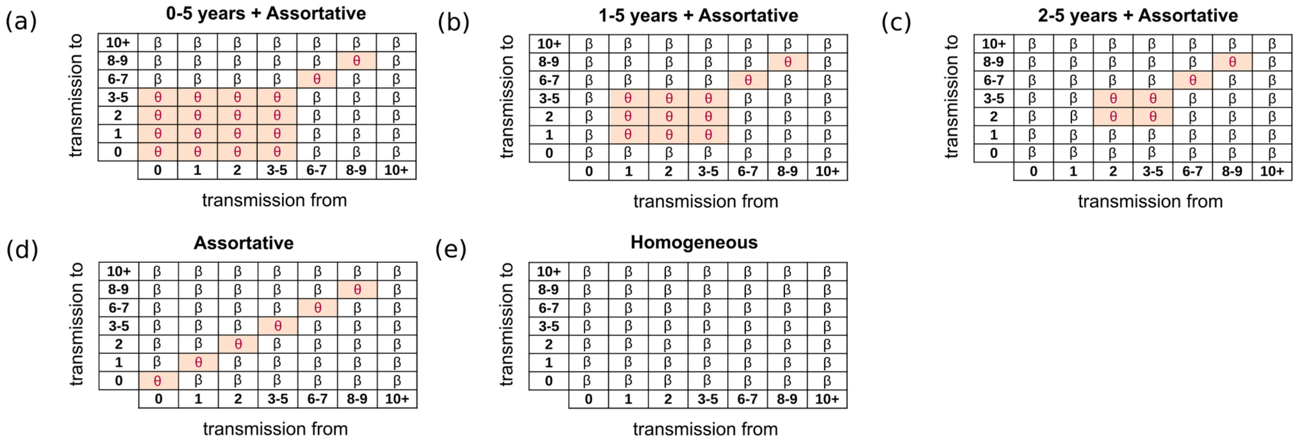

Figure S1: Transmission matrices used for sensitivity of the model. Subplots (a) to (e) present the five transmission matrices used in five independent fitting exercises of the model to the survey data described in the main text. The names of each matrix (and model) is above each subplot. The β and θ letters refer to the transmission coefficients described in the main text. The coefficient θ is in principle larger than β, but this relationship is not explicitly forced in the fitting approach.

The assumption, observation or estimation that pneumococcal transmission is inhomogeneous across ages is common in the literature. For instance, Ojal *et al.* have estimated that the probabilities of acquisition (colonization) across ages in Kilifi, Kenya after the introduction of PCV10, concluding that the probability of the age-group 1-5 years old is about 1.25 and 5.7 times higher than in the age-groups 6-14 and 15-20 years old [20]. For Finland [21], it has been estimated that around 44% of colonization events have a child <7 years of age as their source and that 18% of transmission events occured within this age group; and in Italy, the odds ratio for colonization in children aged <2 years after PCV13 introduction was still high at 3.75 [22]. In a different study by Adetifa *et al.*, carriage was 1.4 times higher in children <4 years of age compared to the next age-group 5-14 years of age [23]. Still in Africa, Uganda, Polain de Waroux *et al.* found that the age-group 5-14 years had the highest frequency of contacts, and a strong age-assortative pattern was seen across age-groups [24]. The latter effect has been reported in several countries including Uganda, Kenya, Finland, USA, and Great Britain [21], [24]–[27]. The epidemiological importance of considering higher efficiency and / or contact numbers between and within younger age-groups is not restricted to the pneumococcus, and is often reported for other pathogens as well [28].

After fitting the model to the survey data in five independent exercises (each with one of the matrices in Figure S1), we compared the frameworks using leave-one-out cross-validation (LOO) and widely applicable information criterion (WAIC) measures. Comparisons were done based on (1) the estimated posteriors versus priors, and (2) posterior model carriage levels versus observed surveys 1 – 7. The priors included were the carriage duration by Hogberg and colleagues [5] (Table S1, Figure 1b) and the vaccine efficacy against carriage (Table S2 and Figure 2c). LOO and WAIC are methods that allow estimating pointwise out-of-sample prediction accuracy from the five models using the log-likelihood evaluated at simulations from the parameter values of the estimated

posteriors [29]. We used the loo R-package [30] to calculate model weights: 1) WAIC weights, 2) Pseudo-BMA weights without Bayesian bootstrap, 3) Pseudo-BMA + weights with Bayesian bootstrap, and 4) Bayesian stacking weights [31]. All of these measures vary between 0 and 1, with the sum between models adding up to 1; the highest the weight, the more favoured a model is compared to the rest. That is, a higher weight points to a more balanced response from the model, both in terms of reproducing the survey data but also in respecting the priors for duration of carriage and the literature knowledge on vaccine efficacy against carriage. The results of model comparison are in Table S3.

**model / matrix**	**1) WAIC**	**2) Pseudo-BMA**	**3) Pseudo-BMA with BS**	**4) Bayesian stacking**
0-5 years +Assortative	0.69	0.99	0.87	0.84
1-5 years + Assortative	0.31	0.01	0.13	0.16
2-5 years + Assortative	0.00	0.00	0.00	0.00
Assortative	0.00	0.00	0.00	0.00
Homogeneous	0.00	0.00	0.00	0.00

Table S3 – LOO and WAIC weights between model frameworks.

The best score was for the model with the 0-5 years + Assortative transmission matrix (Figure 1a). This is therefore the model presented in the main text. In this model, the transmission potential (θ) among individuals 0-5 years old, and among 7-6 and 8-9 years old is different (larger, Figure 2d) than among other age-groups (β). The second best scored model (1-5 years + Assortative, Figure 1b) was the one using the most similar matrix to 0-5 years + Assortative, but which critically did not consider individuals of age 0 years old (i.e. <1 years). For completion, we present the general output of the 1-5 years + Assortative model in Figure S2.

**age-group**	**Carriage reduction after 1 year**
**0**	48.2 (38.7-57.1)
**1**	26.87 (23.1-29.7)
**2**	14.9 (13.4-15.9)
**3-5**	7.3 (6.6-7.8)
**0-5**	16.5 (14.9-17.1)
**6-9**	6.1 (5.6-6.7)

**Vaccine efficacy**
62.28 (46.24-77.60)

**age-group**	**Carriage reduction after 10 years**
**0**	79.8 (67.9-87.7)
**1**	75.1 (65.2-81.7)
**2**	74.3 (64.4-80.7)
**3-5**	74.6 (64.9-80.8)
**0-9**	75.1 (64.5-81.9)

**Pre-vaccination carriage 0-5 years**
51.2 (41.3-59.3)

**age-group**	**Posterior adjustment to priors of carriage duration**
**0-2**	-0.68
**3-5**	+0.58
**6-7**	+0.53
**8+**	-1.71

**age-group**	**10 year FOI reduction**
**0-5**	5.08
**6-7**	6.09
**8-9**	5.93

Table S4 – Posteriors for the model 1-5 years + Assortative. Ranges are the 95% CI.

The 1-5 years + Assortative model presents differences in output and capacity to fit the data. For instance, large differences in the posterior for vaccine efficacy against carriage and the estimation of pre-vacination VT carriage among 0-5 years old can be visually identified comparing Figure 2c,

Figure 5a and Figure S2. For completion, we include here the 1-5 years + Assortative model full range of results (Table S4) presented in the main text for the 0-5 years + Assortative.

Although we defined the model transmission matrices similarly to matrices reported in previous studies, it is likely that further important heterogeneities exist between age-groups. To assess this, however, would require the addition of several parameters to the system, which in turn would increase the complexity and uncertainty of the fitting exercise. It is also the case that without further empirical support, it is difficult to plan and develop this new parametrization, as the required details on which age-group have specific lower / higher transmission coefficients in the particular context of the Blantyre cohort is largely unknown.

The independent contribution of either mixing (e.g. contact frequency) or risk (e.g. efficiency of contact) to the force of infection of each age-group can not be disentangled in our modelling framework. Nonetheless, we note that both higher mixing and risk of infection are supported in our study. For the first, as described above, we resort to literature reports on particular mixing patterns between age groups. For the second, we note that a higher proportion of children 3-6 years of age lived in houses with some lower quality infrastructure and had greater reliance on shared communal water sources in the cohort of our observational study [4].

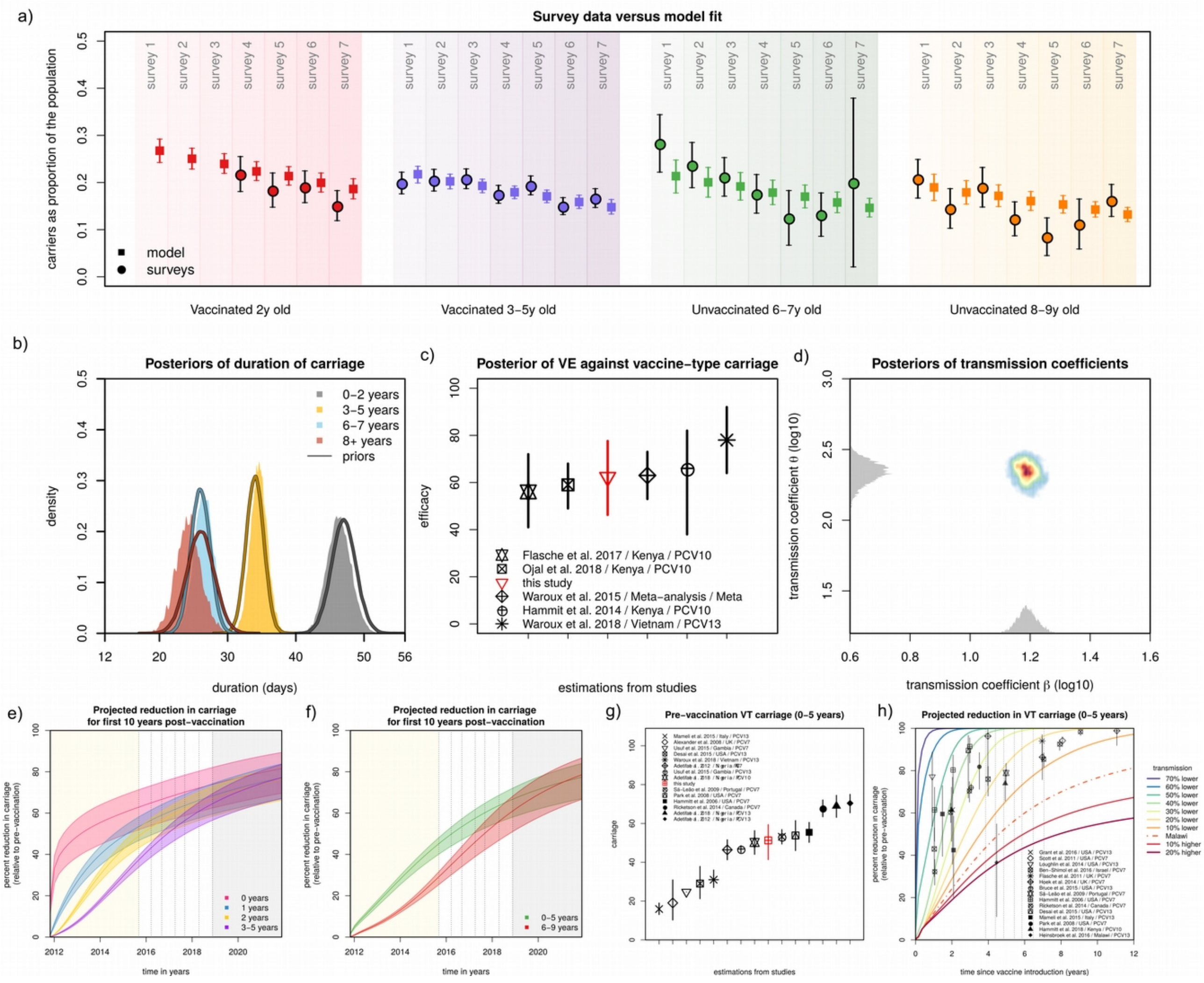

Figure S2: Model fit and estimated posteriors when using a framework that uses the 1-5 years + Assortative transmission matrix. (a) Model fit to carriage data from the observational study for age-groups: vaccinated 2 years old (red), vaccinated 3-5 years old (purple), unvaccinated 6-7 years old (green) and unvaccinated 8-9 years old (orange). The survey data is represented with means as full circles, the model output with means as full squares; the whiskers are the 95% CI. **(b)** Priors (lines) and estimated posterior distributions (shaded areas) for duration of carriage per age-group. **(c)** Estimated posterior for vaccine efficacy (and 95% CI) against vaccine-type carriage (red) in the context of estimations from other studies (in legend, Table S2). **(d)** Estimated posterior for the transmission coefficient β. **(e)** Projected reduction in carriage relative to the pre-vaccination era for age-groups 0 years (magenta), 1 year (blue), 2 years (yellow) and 3-5 years (purple) old. **(f)** Projected reduction in carriage relative to the pre-vaccination era for aggregated age-groups 0-5 years (green) and 6-9 years (red) old (with corresponding 95% CIs). The shaded areas are: yellow for the post-vaccination period with no carriage data, white for the post-vaccination period with survey carriage data, and grey for the post-vaccination projected period up to 10 years. **(g)** Estimated pre-vaccination vaccine-type carriage (and 95% CI) for the age-group 0-5 years of age (red) in the context of carriage levels reported in other studies (in legend, Table S6). **(h)** The baseline transmission coefficient (β) is varied by considering the 70%, 60%, 50%, 40%, 30%, 20%, and 10% lower, and 10%, 20% higher transmission than the estimated for Blantyre (Malawi, β_{Malawi}) when fitting the observational study (e.g. 10% lower is $0.9*\beta_{Malawi}$). The impact projections for the age-group 0-5 years old using the β estimated for Blantyre (Malawi) are presented by the dashed line (as in Figure 3b). The symbols and whiskers are measures of reported impact (carriage reduction) and 95% CIs for several published studies (in legend, Table S5). **(a,b,c,d,e,f,g,h)** Solutions presented are obtained from sampling 100,000 parameter values from posteriors and simulating the dynamic model.

Literature review of carriage reduction in time

The pre- and post-vaccination proportions of vaccine-type carriage were used to calculate a Relative Risk (RR) and its corresponding 95% CI (under asymptotic normality). Using the expression (carriage(post) – carriage(pre)) / carriage(post) = 1 – carriage(pre) / carriage(post) = 1 - RR yielded a corresponding 95% CI for the relative change in carriage (Table S5) for particular time points after vaccination, age-groups, vaccines and countries. These values are used in figures of the main text and other supplementary figures comparing results from Blantyre (Malawi) with other countries.

Percent Reduction	Time	Age	Vaccine	Ref.	Country / Region
80.13% (95% CI 70.31 - 86.71)	3	11 m	PCV7	[36]	Netherlands
69.33% (95% CI 46.65 - 82.37)	4	6–23 m	PCV7	[43]	USA
34.84% (95% CI 24.52 - 43.74)	2	6–24 m	PCV7	[50]	France
52.62% (95% CI 43.6 - 60.2)	3	6–24 m	PCV7	[50]	France
60.88% (95% CI 53.27 - 67.26)	4	6–24 m	PCV7	[50]	France
49.63% (95% CI 33.99 - 61.57)	1	6–24 m	PCV13	[51]	France
95.31% (95% CI 93.44 - 96.65)	5	<2 y	PCV13	[52]	China
88.05% (95% CI 79.64 - 92.99)	3	2 y	PCV7	[36]	Netherlands
77.17% (95% CI 53.76 - 88.73)	1	<5 y	PCV13	[32]	USA
78.91% (95% CI 73.16 - 83.42)	5	<5 y	PCV7	[33]	Portugal
61.44% (95% CI 46.63 - 72.14)	1	<5 y	PCV7	[34]	USA
80.32% (95% CI 69.28 - 87.39)	2	<5 y	PCV7	[34]	USA
91.41% (95% CI 83.65 - 95.49)	3	<5 y	PCV7	[34]	USA
32.24% (95% CI 25.93 - 38.02)	1	<5 y	PCV7	[35]	Canada

60.26% (95% CI 55.48 - 64.52)	2	<5 y	PCV7	[35]	Canada
70.36% (95% CI 65.14 - 74.79)	3	<5 y	PCV7	[35]	Canada
85.35% (95% CI 81.62 - 88.32)	7	<5 y	PCV7	[35]	Canada
92.52% (95% CI 89.6 - 94.61)	8	<5 y	PCV7	[35]	Canada
98.41% (95% CI 96.67 - 99.24)	9	<5 y	PCV7	[35]	Canada
89.56% (95% CI 67.11 - 96.69)	3	<5 y	PCV13	[37]	USA
42.45% (95% CI 20.15 - 58.52)	2	<5 y	PCV13	[38]	Italy
59.5% (95% CI 39.48 - 72.9)	1.5	<5 y	PCV13	[38]	Italy
81.82% (95% CI 71.19 - 88.52)	3.5	<5 y	PCV7	[39]	USA
94% (95% CI 84 - 97)	7	<5 y	PCV7	[40]	UK
43% (95% CI 30 - 53)	1	<5 y	PCV7	[41]	Israel
76% (95% CI 68 - 82)	4	<5 y	PCV7	[41]	Israel
86.36% (95% CI 75.78 - 92.32)	7	<5 y	PCV7	[42]	England
98.86% (95% CI 91.91 - 99.84)	11	<5 y	PCV7	[42]	England
96.3% (95% CI 72.97 - 99.49)	4	<5 y	PCV13	[42]	England
71.98% (95% CI 70.15 - 73.71)	3	<5 y	PCV13	[44]	USA
61.46% (95% CI 35.75 - 76.88)	2	<5 y	PCV13	[45]	USA
94.17% (95% CI 91.48 - 96.01)	8	<5 y	PCV7	[46]	USA
73.92% (95% CI 75.73 – 80.12)	5	<5 y	PCV10	[47]	Kenya
61.45% (95% CI 46.62 – 72.16)	2	<5 y	PCV10	[47]	Kenya
68.84% (95% CI 43.6 - 82.78)	4	2 to <7 y	PCV7	[43]	USA
49.16% (95% CI 28.24 - 63.98)	1.5	>15 y	PCV7	[48]	Australia
36.57% (95% CI 10.97 - 54.81)	4.5	1-4 y	PCV13	[49]	Malawi

Table S5 – Percent reduction in carriage for several studies. Mean, 95% CI, age-group, time since vaccination, vaccine, reference and country are presented for each study. See text for mean and CI calculation.

Literature review of pre-vaccination carriage levels

Table S6 presents a literature review of pre-vaccination carriage levels for different groups of (vaccine) serotypes in different epidemiological contexts. These values are used in figures of the main text comparing results from Blantyre (Malawi) with other countries.

Carriage in %	Age	Vaccine types	Ref.	Country / Region
53.80% (95% CI 46.02 - 61.44)	<5 y	PCV7	[39]	USA
27% (95% CI 23 - 32)	<5 y	PCV13	[18]	Vietnam
67.87% (95% CI 63.44 - 72.07)	<5 y	PCV7	[35]	Canada
24.7% (95% CI 24.7 - 24.7)	<5 y	PCV7	[53]	Gambia
53.13% (95% CI 49.40 - 56.84)	<5 y	PCV7	[33]	Portugal
55.43% (95% CI 50.26 - 60.52)	<5 y	PCV7	[34]	USA
19.04% (95% CI 10.24 - 30.90)	<5 y	PCV7	[55]	UK
29.03% (95% CI 21.23 - 37.86)	<5 y	PCV13	[37]	USA
15.9% (95% CI 13.06 - 18.98)	<5 y	PCV13	[38]	Italy
46.8% (95% CI 46.8 - 46.8)	<5 y	PCV13	[53]	Gambia
46.4% (95% CI 41.26 - 51.59)	<5 y	PCV7	[23]	Nigeria
70.4% (95% CI 65.49 - 74.97)	<5 y	PCV13	[23]	Nigeria

50.2% (95% CI 44.07 - 56.29)	<5 y	PCV10	[54]	Nigeria
69% (95% CI 63.12 - 74.45)	<5 y	PCV13	[54]	Nigeria

Table S6 – Pre-vaccination carriage levels for several studies. Mean, 95% CI, age-group, vaccine types (serotypes of particular PCV vaccine), reference and country are presented for each study.

Extra results for sensitivity of vaccine impact projections

In Figures S3 and S4 we present extra results on the sensitivity of vaccine impact projections relative to baseline transmission, as an extension to main Figure 5 (and thus using the transmission matrix of Figure S1a. Figure S3 is based on all age-groups under the age of 10 years old as projected by the model (for comparison, the age-groups of main Figure 5 are presented again). Figure S4 presents two age classes for which we found empirical reports on carriage decrease post-vaccination, as an extension to main Figure 5.

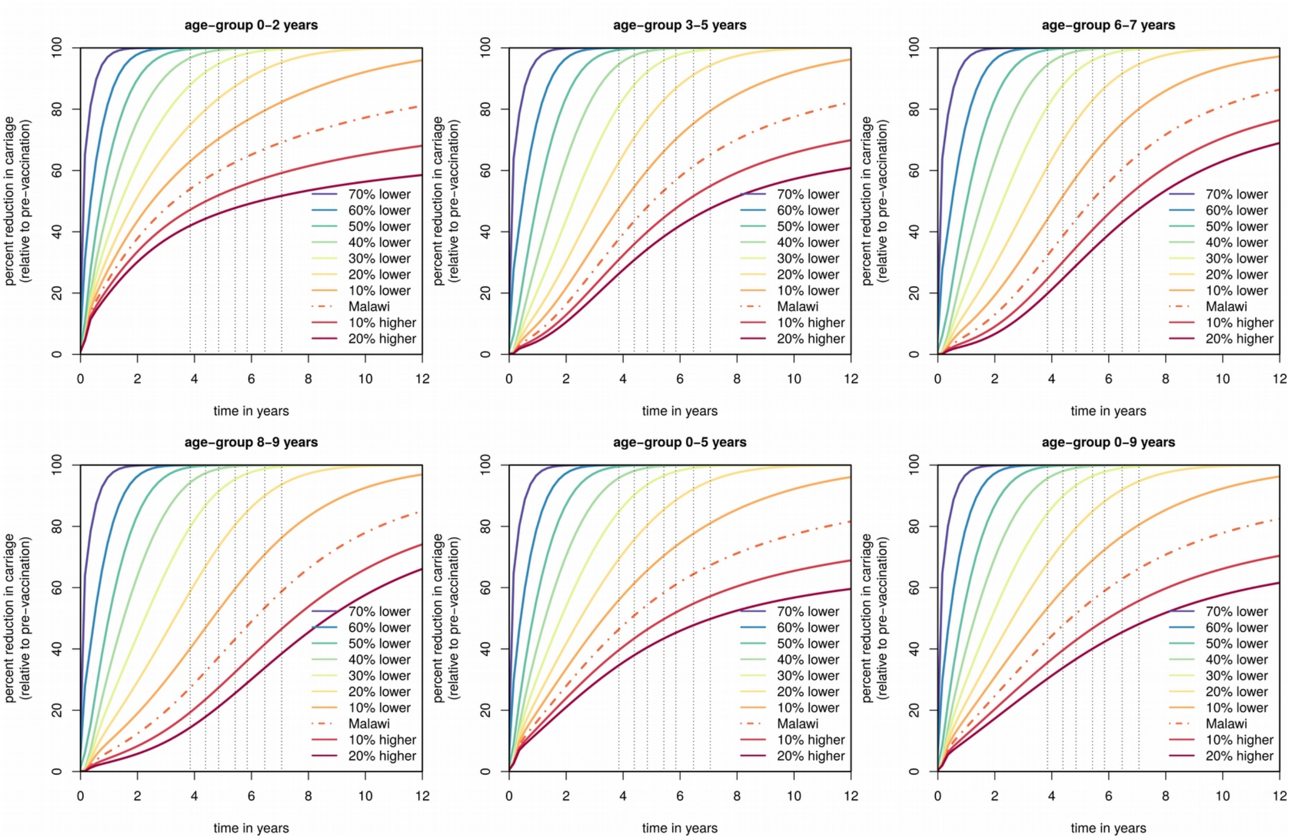

Figure S3: Extra results for sensitivity of impact projections to baseline transmission (model age-groups under the age of 10 years). Figure 5 of the main text presents results for age-group 0-5 years of age. This figure presents results for all age-groups. The baseline transmission is varied by considering the 70%, 60%, 50%, 40%, 30%, 20%, 10% lower, and 10%, 20%, 30% higher relative change to the original values estimated for β and θ when fitting the observational study's data (e.g. 40% lower means 0.6*β and 0.6*θ). The impact projections using β and θ of Blantyre (Malawi) are presented with the shaded line (same as in main text). For visual purposes, only the means are shown, obtained from simulations sampling 100,000 parameter values from posteriors.

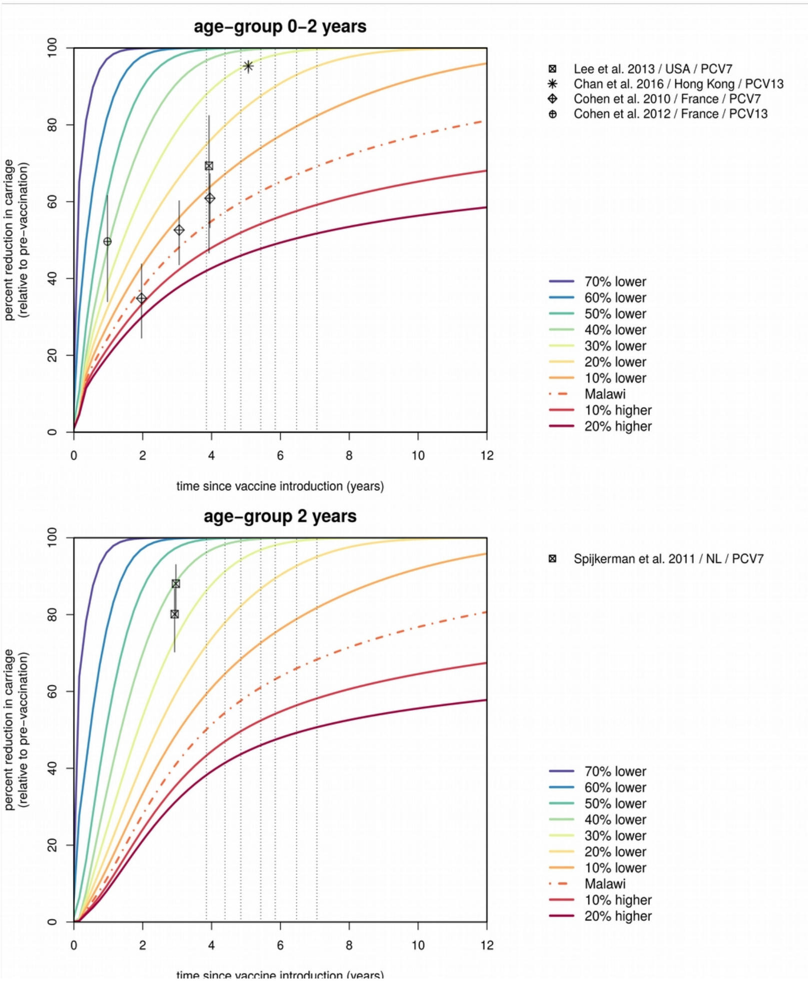

Figure S4: Extra results for sensitivity of impact projections to baseline transmission (two model age-groups with empirical observations). Figure 5 of the main text presents results for age-group 0-5 years of age including reported data. This figure presents results for two other age-groups for which reports were available. The baseline transmission is varied by considering the 70%, 60%, 50%, 40%, 30%, 20%, 10% lower, and 10%, 20%, 30% higher relative change to the original values estimated for β and θ when fitting the observational study's data (e.g. 40% lower means 0.6*β and 0.6*θ). The impact projections using β and θ of Blantyre (Malawi) are presented with the shaded line (same as in main text). For visual purposes, only the means are shown, obtained from simulations sampling 100,000 parameter values from posteriors.

Observational study data

Table S7 keeps the carriage levels observed in the observational surveys and used to fit the model.

**survey \ age-group**	Vacc. 2 years	Vacc. 3-5 years	Unvacc. 6-7 years	Unvacc. 8-9 years
Survey 1	No data	0.199 (0.0236)	0.283 (0.0619)	0.208 (0.0414)
Survey 2	No data	0.205 (0.0232)	0.237 (0.0488)	0.145 (0.0424)
Survey 3	No data	0.208 (0.0214)	0.212 (0.0411)	0.19 (0.0428)
Survey 4	0.218 (0.0371)	0.175 (0.0196)	0.176 (0.0413)	0.123 (0.0366)
Survey 5	0.184 (0.0363)	0.194 (0.0205)	0.125 (0.0585)	0.0851 (0.0407)
Survey 6	0.191 (0.0337)	0.15 (0.0183)	0.132 (0.0465)	0.112 (0.0353)
Survey 7	0.151 (0.0319)	0.167 (0.0207)	0.2 (0.179)	0.162 (0.0341)

Table S7 – Carriage data from the observational study (mean and standard error)

References

[1] J. Lourenço *et al.*, “Epidemiological and ecological determinants of Zika virus transmission in an urban setting.,” *Elife*, vol. 6, Sep. 2017.

[2] A. McNaughton *et al.*, “Utilising a Cohort Study of Hepatitis B Virus (HBV) Vaccine-Mediated Immunity in South African Children to Model Infection Dynamics: Can We Meet Global Targets for Elimination by 2030?,” *bioRxiv*, Jul. 2017.

[3] N. R. Faria *et al.*, “Genomic and epidemiological characterisation of a dengue virus outbreak among blood donors in Brazil,” *Sci. Rep.*, vol. 7, no. 1, p. 15216, Dec. 2017.

[4] T. D. Swarthout *et al.*, “High residual prevalence of vaccine-serotype Streptococcus pneumoniae carriage after introduction of a pneumococcal conjugate vaccine in Malawi: a prospective serial cross-sectional study,” *bioRxiv*, 2019.

[5] L. Hogberg, P. Geli, H. Ringberg, E. Melander, M. Lipsitch, and K. Ekdahl, “Age- and Serogroup-Related Differences in Observed Durations of Nasopharyngeal Carriage of Penicillin-Resistant Pneumococci,” *J. Clin. Microbiol.*, vol. 45, no. 3, pp. 948–952, Mar. 2007.

[6] K. Ekdahl, H. B. Hansson, S. Mölstad, M. Söderström, M. Walder, and K. Persson, “Duration of Nasopharyngeal Carriage of Penicillin-Resistant Streptococcus pneumoniae: Experiences from the South Swedish Pneumococcal Intervention Project,” *Microb. Drug Resist.*, vol. 4, no. 2, pp. 99–105, 1998.

[7] P. Turner *et al.*, “A longitudinal study of streptococcus pneumoniae carriage in a cohort of infants and their mothers on the Thailand-Myanmar border,” *PLoS One*, vol. 7, no. 5, 2012.

[8] O. Abdullahi *et al.*, “Rates of acquisition and clearance of pneumococcal serotypes in the nasopharynges of children in Kilifi District, Kenya,” *J. Infect. Dis.*, vol. 206, no. 7, pp. 1020–1029, 2012.

[9] F. Hoti, P. Erästö, T. Leino, and K. Auranen, “Outbreaks of Streptococcus pneumoniae carriage in day care cohorts in Finland - Implications for elimination of transmission,” *BMC Infect. Dis.*, vol. 9, pp. 1–13, 2009.

[10] P. Erästö, F. Hoti, S. M. Granat, Z. Mia, P. H. Mäkelä, and K. Auranen, “Modelling multi-type transmission of pneumococcal carriage in Bangladeshi families,” *Epidemiol. Infect.*, vol. 138, no. 6, pp. 861–872, 2010.

[11] A. Melegaro, Y. Choi, R. Pebody, and N. Gay, “Pneumococcal carriage in United Kingdom families: Estimating serotype-specific transmission parameters from longitudinal data,” *Am. J. Epidemiol.*, vol. 166, no. 2, pp. 228–235, 2007.

[12] A. Melegaro, Y. H. Choi, R. George, W. J. Edmunds, E. Miller, and N. J. Gay, “Dynamic models of pneumococcal carriage and the impact of the Heptavalent Pneumococcal

Conjugate Vaccine on invasive pneumococcal disease," *BMC Infect. Dis.*, vol. 10, no. 1, p. 90, 2010.

[13] A. Melegaro, N. J. Gay, and G. F. Medley, "Estimating the transmission parameters of pneumococcal carriage in households," *Epidemiol. Infect.*, vol. 132, no. 3, pp. 433–441, 2004.

[14] K. Ekdahl *et al.*, "Duration of Nasopharyngeal Carriage of Penicillin   Resistant Streptococcus pneumoniae : Experiences from the South Swedish Pneumococcal Intervention Project," *Clin. Infect. Dis.*, vol. 25, no. 5, pp. 1113–1117, Nov. 1997.

[15] S. Flasche *et al.*, "Assessing the efficiency of catch-up campaigns for the introduction of pneumococcal conjugate vaccine: A modelling study based on data from PCV10 introduction in Kilifi, Kenya," *BMC Med.*, vol. 15, no. 1, pp. 1–10, 2017.

[16] L. L. Hammitt *et al.*, "Population effect of 10-valent pneumococcal conjugate vaccine on nasopharyngeal carriage of Streptococcus pneumoniae and non-typeable Haemophilus influenzae in Kilifi, Kenya: Findings from cross-sectional carriage studies," *Lancet Glob. Heal.*, vol. 2, no. 7, pp. e397–e405, 2014.

[17] O. Le Polain De Waroux, S. Flasche, D. Prieto-Merino, D. Goldblatt, and W. J. Edmunds, "The efficacy and duration of protection of pneumococcal conjugate vaccines against nasopharyngeal carriage: A meta-regression model," *Pediatr. Infect. Dis. J.*, vol. 34, no. 8, pp. 858–864, 2015.

[18] O. Le Polain de Waroux *et al.*, "Predicting the impact of pneumococcal conjugate vaccine programme options in Vietnam," *Hum. Vaccin. Immunother.*, vol. 0, no. 0, pp. 1–21, 2018.

[19] J. Ojal *et al.*, "The merits of sustaining pneumococcal vaccination after transitioning from Gavi support - a modelling and cost-effectiveness study for Kenya," *bioRxiv*, Jan. 2018.

[20] J. Ojal *et al.*, "Sustained reduction in vaccine-type invasive pneumococcal disease despite waning effects of a catch-up campaign in Kilifi, Kenya: A mathematical model based on pre-vaccination data," *Vaccine*, vol. 35, no. 35, pp. 4561–4568, 2017.

[21] M. Nurhonen, A. C. Cheng, and K. Auranen, "Pneumococcal Transmission and Disease In Silico: A Microsimulation Model of the Indirect Effects of Vaccination," *PLoS One*, vol. 8, no. 2, 2013.

[22] R. Camilli *et al.*, "Pneumococcal Carriage in Young Children One Year after Introduction of the 13-Valent Conjugate Vaccine in Italy," *PLoS One*, vol. 8, no. 10, pp. 1–10, 2013.

[23] I. M. O. Adetifa *et al.*, "Pre-vaccination nasopharyngeal pneumococcal carriage in a Nigerian population: Epidemiology and population biology," *PLoS One*, vol. 7, no. 1, 2012.

[24] O. le Polain de Waroux *et al.*, "Characteristics of human encounters and social mixing patterns relevant to infectious diseases spread by close contact: A survey in Southwest Uganda," *BMC Infect. Dis.*, vol. 18, no. 1, pp. 1–12, 2018.

[25] B. M. Althouse *et al.*, “Identifying transmission routes of Streptococcus pneumoniae and sources of acquisitions in high transmission communities,” *Epidemiol. Infect.*, vol. 145, no. 13, pp. 2750–2758, 2017.

[26] J. Mossong *et al.*, “Social contacts and mixing patterns relevant to the spread of infectious diseases,” *PLoS Med.*, vol. 5, no. 3, pp. 0381–0391, 2008.

[27] M. C. Kiti, T. M. Kinyanjui, D. C. Koech, P. K. Munywoki, G. F. Medley, and D. J. Nokes, “Quantifying age-related rates of social contact using diaries in a rural coastal population of Kenya,” *PLoS One*, vol. 9, no. 8, 2014.

[28] K. T. D. Eames, N. L. Tilston, E. Brooks-Pollock, and W. J. Edmunds, “Measured dynamic social contact patterns explain the spread of H1N1v influenza,” *PLoS Comput. Biol.*, vol. 8, no. 3, pp. 1–8, 2012.

[29] A. Vehtari, A. Gelman, and J. Gabry, “Practical Bayesian model evaluation using leave-one-out cross-validation and WAIC,” *Stat. Comput.*, vol. 27, no. 5, pp. 1–20, 2016.

[30] A. Vehtari, J. Gabry, Y. Yao, and A. Gelman, “loo: Efficient leave-one-out cross-validation and WAIC for Bayesian models.” 2018.

[31] Y. Yao, A. Vehtari, D. Simpson, and A. Gelman, “Using Stacking to Average Bayesian Predictive Distributions,” *Bayesian Anal.*, pp. 1–74, Jan. 2018.

[32] A. M. Loughlin, K. Hsu, A. L. Silverio, C. D. Marchant, and S. I. Pelton, “Direct and indirect effects of PCV13 on nasopharyngeal carriage of PCV13 unique pneumococcal serotypes in Massachusetts’ children,” *Pediatr. Infect. Dis. J.*, vol. 33, no. 5, pp. 504–510, 2014.

[33] R. Sá-Leão *et al.*, “Changes in pneumococcal serotypes and antibiotypes carried by vaccinated and unvaccinated day-care centre attendees in Portugal, a country with widespread use of the seven-valent pneumococcal conjugate vaccine,” *Clin. Microbiol. Infect.*, vol. 15, no. 11, pp. 1002–1007, 2009.

[34] L. L. Hammitt *et al.*, “Indirect Effect of Conjugate Vaccine on Adult Carriage of *Streptococcus pneumoniae:* An Explanation of Trends in Invasive Pneumococcal Disease,” *J. Infect. Dis.*, vol. 193, no. 11, pp. 1487–1494, 2006.

[35] L. J. Ricketson *et al.*, “Trends in asymptomatic nasopharyngeal colonization with streptococcus pneumoniae after introduction of the 13-valent pneumococcal conjugate vaccine in Calgary, Canada,” *Pediatr. Infect. Dis. J.*, vol. 33, no. 7, pp. 724–730, 2014.

[36] J. Spijkerman *et al.*, “Carriage of *Streptococcus pneumoniae* 3 Years after Start of Vaccination Program, the Netherlands,” *Emerg. Infect. Dis.*, vol. 17, no. 4, pp. 584–591, 2011.

[37] A. P. Desai *et al.*, “Decline in pneumococcal nasopharyngeal carriage of vaccine serotypes after the introduction of the 13-valent pneumococcal conjugate vaccine in children in Atlanta, Georgia,” *Pediatr. Infect. Dis. J.*, vol. 34, no. 11, pp. 1168–1174, 2015.

[38] C. Mameli *et al.*, “A longitudinal study of streptococcus pneumoniae carriage in healthy children in the 13-valent pneumococcal conjugate vaccine era,” *Hum. Vaccin. Immunother.*, vol. 11, no. 4, pp. 811–817, 2015.

[39] S. Y. Park *et al.*, “Impact of conjugate vaccine on transmission of antimicrobial-resistant Streptococcus pneumoniae among Alaskan children,” *Pediatr. Infect. Dis. J.*, vol. 27, no. 4, pp. 335–340, 2008.

[40] S. Flasche *et al.*, “Effect of pneumococcal conjugate vaccination on serotype-specific carriage and invasive disease in England: A cross-sectional study,” *PLoS Med.*, vol. 8, no. 4, p. 14, 2011.

[41] S. Ben-Shimol, N. Givon-Lavi, D. Greenberg, and R. Dagan, “Pneumococcal nasopharyngeal carriage in children <5 years of age visiting the pediatric emergency room in relation to PCV7 and PCV13 introduction in southern Israel,” *Hum. Vaccines Immunother.*, vol. 12, no. 2, pp. 268–276, 2016.

[42] A. J. Van Hoek *et al.*, “Pneumococcal carriage in children and adults two years after introduction of the thirteen valent pneumococcal conjugate vaccine in England,” *Vaccine*, vol. 32, no. 34, pp. 4349–4355, 2014.

[43] G. M. Lee *et al.*, “Impact of 13-valent pneumococcal conjugate vaccination on Streptococcus pneumoniae carriage in young children in Massachusetts,” *J. Pediatric Infect. Dis. Soc.*, vol. 3, no. 1, pp. 23–32, 2014.

[44] M. G. Bruce *et al.*, “Impact of the 13-valent pneumococcal conjugate vaccine (pcv13) on invasive pneumococcal disease and carriage in Alaska,” *Vaccine*, vol. 33, no. 38, pp. 4813–4819, 2015.

[45] L. R. Grant *et al.*, “Impact of the 13-Valent Pneumococcal Conjugate Vaccine on Pneumococcal Carriage Among American Indians,” *Pediatr. Infect. Dis. J.*, vol. 35, no. 8, pp. 907–914, Aug. 2016.

[46] J. R. Scott *et al.*, “Impact of more than a decade of pneumococcal conjugate vaccine use on carriage and invasive potential in native American communities,” *J. Infect. Dis.*, vol. 205, no. 2, pp. 280–288, 2012.

[47] L. L. Hammitt *et al.*, “Effect of ten-valent pneumococcal conjugate vaccine on invasive pneumococcal disease and nasopharyngeal carriage in Kenya: a longitudinal surveillance study,” *Lancet*, vol. 393, no. 10186, pp. 2146–2154, 2019.

[48] D. A. Collins *et al.*, “Predictors of pneumococcal carriage and the effect of the 13-valent pneumococcal conjugate vaccination in the Western Australian Aboriginal population,” *Pneumonia*, vol. 9, no. 1, p. 14, 2017.

[49] E. Heinsbroek, “Pneumococcal carriage and transmission in Karonga district , Malawi , before and after introduction of 13-valent pneumococcal conjugate vaccination,” University of Liverpool, 2016.

[50] R. Cohen *et al.*, “Dynamic of pneumococcal nasopharyngeal carriage in children with acute otitis media following PCV7 introduction in France,” *Vaccine*, vol. 28, no. 37, pp. 6114–6121, 2010.

[51] R. Cohen, C. Levy, E. Bingen, M. Koskas, I. Nave, and E. Varon, “Impact of 13-valent Pneumococcal Conjugate Vaccine on Pneumococcal Nasopharyngeal Carriage in Children With Acute Otitis Media,” *Pediatr. Infect. Dis. J.*, vol. 31, no. 3, pp. 297–301, 2012.

[52] K. C. C. Chan *et al.*, “Pneumococcal carriage in young children after introduction of PCV13 in Hong Kong,” *Vaccine*, vol. 34, no. 33, pp. 3867–3874, 2016.

[53] E. Usuf *et al.*, “Pneumococcal carriage in rural Gambia prior to the introduction of pneumococcal conjugate vaccine: A population-based survey,” *Trop. Med. Int. Heal.*, vol. 20, no. 7, pp. 871–879, 2015.

[54] I. M. O. Adetifa *et al.*, “Nasopharyngeal Pneumococcal Carriage in Nigeria: A two-site, population-based survey,” *Sci. Rep.*, vol. 8, no. 1, pp. 1–9, 2018.

[55] E. Alexander, P. Telfer, H. Rashid, K. A. Ali, and R. Booy, “Nasopharyngeal carriage rate of Streptococcus pneumoniae in children with sickle cell disease before and after the introduction of heptavalent pneumococcal conjugate vaccine,” *J. Infect. Public Health*, vol. 1, no. 1, pp. 40–44, 2008.
